# Design, Synthesis and Actual Applications of the Polymers Containing Acidic P–OH Fragments: Part 1. Polyphosphodiesters

**DOI:** 10.3390/ijms232314857

**Published:** 2022-11-28

**Authors:** Ilya E. Nifant’ev, Pavel V. Ivchenko

**Affiliations:** 1 A.V. Topchiev Institute of Petrochemical Synthesis RAS, 29 Leninsky Pr., 119991 Moscow, Russia; 2 Chemistry Department, M.V. Lomonosov Moscow State University, 1–3 Leninskie Gory, 119991 Moscow, Russia

**Keywords:** biocompatibility, biodegradable polymers, biomineralization, drug delivery, metathesis polymerization, polycondensation, polymer composites, polyphosphates, poly(phosphodiester)s, polyphosphonates, phosphodiesters, ring-opening polymerization

## Abstract

Among natural and synthetic polymers, main-chain phosphorus-containing polyacids (PCPAs) (polyphosphodiesters), stand in a unique position at the intersection of chemistry, physics, biology and medicine. The structural similarity of polyphosphodiesters PCPAs to natural nucleic and teichoic acids, their biocompatibility, mimicking to biomolecules providing the ‘stealth effect’, high bone mineral affinity of polyphosphodiesters resulting in biomineralization at physiological conditions, and adjustable hydrolytic stability of polyphosphodiesters are the basis for various biomedical, industrial and household applications of this type of polymers. In the present review, we discuss the synthesis, properties and actual applications of polyphosphodiesters.

## 1. Introduction

**Scheme 1:**
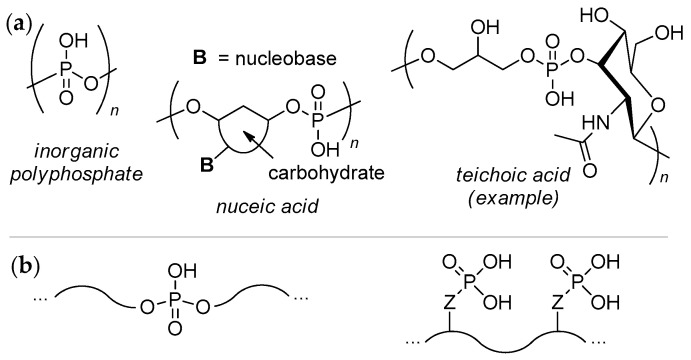
(**a**) Natural phosphorus-containing polyacids (PCPAs); (**b**) Two main types of synthetic phosphorus-containing polyacids (PCPAs).

Over the past few years, synthetic polymers containing acidic phosphate groups have been the subject of extensive research [1,2,3,4,5,6,7]. Their similarity to environmental inorganic polyphosphates [8,9], nucleic acids [10] and teichoic acids (TAs) [11] (Scheme 1a), as well as the biocompatibility of the phosphate group [5,12], offers great opportunities for the use of these polymers for different biomedical [1,2,5,13,14,15,16,17], industrial [4,18] and household [19] applications.

There are two fundamentally different types of phosphorus-containing polyacids (PCPAs). The structure of the first, the closest to natural, type of PCPA implies phosphate fragments in a polymer backbone (main-chain PCPAs, known polymers of this type represent polyphosphodiesters, Scheme 1b). The second type, side-chain PCPAs, represent macromolecules containing acidic phosphate or phosphonate fragments as substituents distributed throughout the polymer backbone (Scheme 1b). The synthetic approaches to these two types of PCPAs are essentially diverse. The areas of application of the PCPAs are also dependent on the position of the phosphorus-containing groups inside or outside the main polymer chain.

The present review comprises critical analysis of the synthetic approaches to the main-chain PCPAs, polyphosphodiesters, and a brief discussion of their properties and actual applications. Repetitive enzymatic syntheses of the close analogs of nucleic acids, reviewed by Jones [20], and acyclic artificial nucleic acids, reviewed by Kashida and coll [21], are not discussed in this work.

Concluding the introduction, we need to address a general issue related to the chemical nomenclature of the phosphorus-containing organic acids and esters. The point is that the compounds of the formula (RO)_2_P(O)H in many works, especially works that have been published for a long time, are termed as ‘phosphites’ (and similar names still persist as a trade names of chemical reagents, e.g., ‘diethyl phosphite’ for (EtO)_2_P(O)H). In our review, we were content to follow the rules of the International Union of Pure and Applied Chemistry (IUPAC) that recommend the attribution of (RO)_2_P(O)(H/R) to ‘phosphonates’, (RO)_2_P(O)(OH/OR) to ‘phosphates’, and (RO)_3_P to ‘phosphites’. Additionally, note that when referring to an article in the present review, we indicated the surname of the corresponding author of the work which does not always coincide with the surname of the research team leader.

## 2. Design and Synthesis of Polyphosphodiesters 

### 2.1. Synthetic Approaches to Polyphosphodiesters: An Overview

**Scheme 2:**
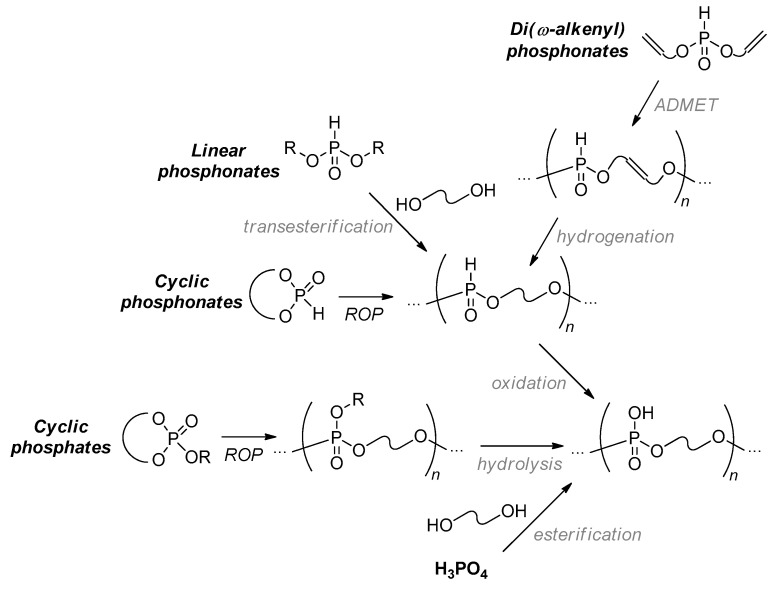
General synthetic approaches to polyphosphodiesters.

In a recent review [5], Iwasaki presented several important examples of the synthetic approaches to PCPAs. In this section, we have tried to enhance, refine and discuss alternative synthetic approaches to polyphosphodiesters. The synthesis of the most simple polyphosphodiesters, poly(ethylene phosphoric acid) (PEPA) and poly(1,3-propylene phosphoric acid) (1,3-PPPA), was reported by Penczek’ group back in 1976 [22]. To date, multiple approaches to polyphosphodiesters have been developed. The most evident synthetic pathway is based on the interaction of phosphoric acid with diols reviewed by Penczek et al. in 2015 [1] or on transesterification of dialkyl (or diaryl) phosphonates followed by oxidation of P–H bonds [23,24,25,26,27,28,29]. Ring-opening polymerization (ROP) of strained cyclic phosphonates (containing P–H bonds) and phosphates, followed by post-modification (oxidation or hydrolysis/hydrolytic thermolysis, respectively) is another efficient pathway to polyphosphodiesters [30,31]. Meanwhile, modern methods of the construction of hydrocarbon fragments of the PCPA backbone, i.e., metathesis polycondensation and polymerization [32,33,34,35,36], should not be dismissed (Scheme 2). Note that the use of acyclic diene metathesis (ADMET) polycondensation in the synthesis of ‘precision polymers’ was the subject of review by Schulz and Wagener [37]. 

### 2.2. Polycondensation and Related Methods

#### 2.2.1. Reactions of H_3_PO_4_ with Diols and Polyols

Phosphoric acid H_3_PO_4_ is a relatively weak tribasic acid (pK_a_^1^ = 2.15, pK_a_^2^ = 7.09, pK_a_^3^ = 12.32). With the transition to pyrophosphoric acid H_4_P_2_O_7_, one can note a substantial increase of acidity (pK_a_^1^ = 1.0, pK_a_^2^ = 2.0) and, therefore, reactivity of H_4_P_2_O_7_ in comparison with H_3_PO_4_. Poly(phosphoric acid) is a well-known ‘superacid’; however, its use in the synthesis of PCPAs is essentially restricted by the requirements of the hydrolytic stability of PCPAs that implies the absence of di-/oligophosphate fragments in the main polymer chain. In this way, successful synthesis of PCPAs was limited by the use of H_3_PO_4_ and H_4_P_2_O_7_ in polycondensation with diols and polyols. This approach was developed mainly by Penczek and coll who studied direct condensation of H_3_PO_4_ with ethylene glycol [38,39,40]. The following steps were detected during this reaction:

- The reaction starts by the relatively slow dimerization of H_3_PO_4_ with a formation of H_4_P_2_O_7_ (and higher polyphosphoric acids) at 100 °C within 40 h, during this stage the water was removed either in the stream of neutral gas or azeotropically with heptane.
- After the addition of EG at 100 °C, H_4_P_2_O_7_ transformed to H_3_PO_4_ immediately, and the first phosphorylation reaction within additional 80 h was the formation of HOCH_2_CH_2_OP(O)(OH)_2_ and (HOCH_2_CH_2_O)_2_P(O)OH, triesters were formed in minimal amounts.
- Activation of the monophosphate esters (end groups) at any polymerization degree with H_3_PO_4_ proceeds via conversion of monoesters into pyrophosphoric acid esters –OCH_2_CH_2_OP(O)(OH)–OP(O)(OH)_2_ that represent reactive acidic sites.
- The polycondensation product is mostly linear with a structure of PEPA –(OCH_2_CH_2_OP(O)(OH))*_n_*–.
- Some branch points (triesters) are formed only at high temperature and prolonged polycondensation time.

The reaction resulted in the formation of relatively low molecular weight (MW) products, the maximum achieved degree of polymerization (*DP*_n_) was 21 after 100 h at 150 °C even in the presence of Sc(CF_3_SO_3_)_3_ as a catalyst. Polycondensation was also accompanied by the formation of ether bonds (di- and triethylene glycol fragments were detected), acetaldehyde and vinyl end-fragments [38,39].

**Scheme 3:**
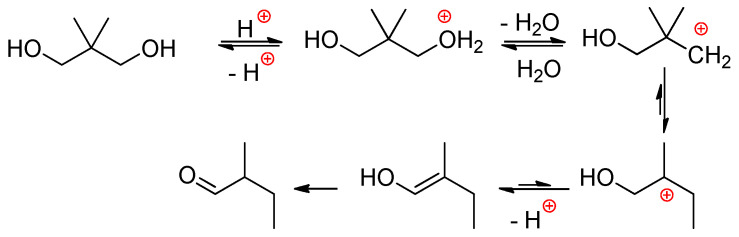
Formation of 2-methylbutanal during the reaction of neopentyl glycol with H_3_PO_4_ [38].

To avoid dehydration side processes during the reaction with H_3_PO_4_, Penczek et al. proposed the use of 2,2-dimethyl-1,3-propanediol; however, no polymers were obtained, and the main reaction product was 2-methylbutanal formed via methyl migration (Scheme 3) [38]. 

The reaction of H_3_PO_4_ with glycerol is a more complex process [40,41,42]. This reaction was conducted at 100 °C with azeotropic water removal (heptane) or under reduced pressure. The rate of esterification and the product ratios depended on the reagent ratios. So, for example, for a H_3_PO_4_/glycerol ratio of 1:1 the conversion of H_3_PO_4_ reached 90% after 35 h, whereas at a H_3_PO_4_/glycerol ratio of 1:2 even after 140 h only 80% conversion was detected, and the ratio of 2:1 led to monoester as a main product, 15% of H_3_PO_4_ remained unreacted. Five- and six-membered cyclic esters were detected in the reaction mixtures in minor amounts. At a 1:1 H_3_PO_4_/glycerol ratio, cross-linking was observed. The degree of polymerization of soluble products was limited by dealkylation, leading to the formation of di- and oligo-glycerol units, incorporated into the product structure. 

Polycondensation of diglycerol (HOCH_2_CH(OH)CH_2_)_2_O) with H_3_PO_4_ resulted in the formation of highly branched gels [42]. The prospects of the further use of these polymers still remains unclear due to the unpredictability of their microstructure and hydrolytic behavior. 

In conclusion, it should be mentioned that the reaction of H_3_PO_4_ with ethylene carbonate, first described by Munoz et al. [43] and reproduced by Imoto and coll [44], resulted in low-MW PEPA with an unknown structure. Additionally, note that the reaction of H_3_PO_4_ with oxirans results in a formation of triester species [45,46] and therefore cannot be considered as a method of the synthesis of PCPAs.

#### 2.2.2. The Reaction of Dichlorophosphates with Diols

**Scheme 4:**
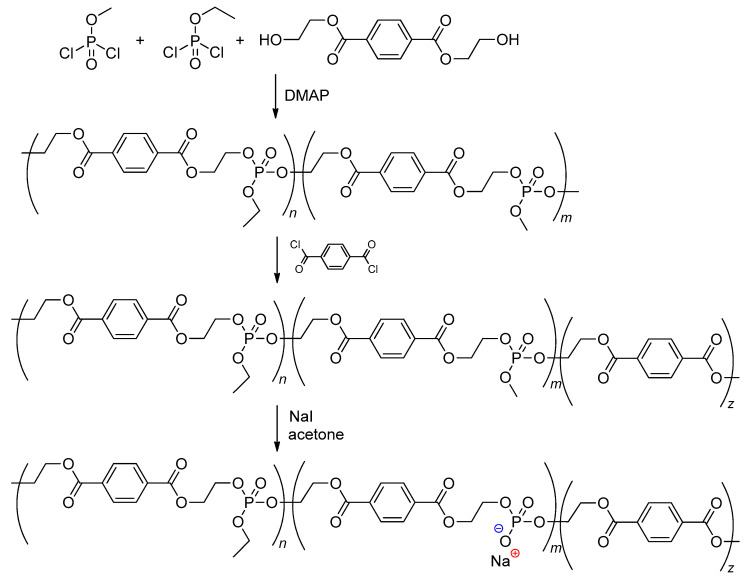
Synthesis of the phosphate-containing analog of PET [49].

Glycolysis of PET with a formation of bis(2-hydroxyethyl)phthalate is the most efficient method of chemical recycling of this polymer [47,48]. The reaction of bis(2-hydroxyethyl)phthalate with Cl_2_P(O)OR (R = Me, Et) resulted in the formation of copolymers, further treatment by terephthaloyl chloride and NaI/acetone allowed for a copolymer containing >P(O)–OH fragments to be obtained [49] (Scheme 4). However, the current trends in developing actual synthetic approaches to biodegradable materials imply the abandonment of chlorine-containing reagents, and therefore dichlorophosphates are not currently used in the synthesis of polyphosphodiesters. 

#### 2.2.3. Reaction of Dialkyl (or Diaryl) Phosphonates with Diols and Post-Modification

Since polymers with –O–P(O)H–O– fragments can be easily and almost quantitatively oxidized to corresponding poly(phosphodiesters) containing –O–P(O)(OH)–O– fragments [22,23,50], polycondensation of dialkyl phosphonates (RO)_2_P(O)H with diols can be considered as a prospective method of the synthesis of polyphosphodiesters. However, when using propane-1,3-diol, a six-membered cyclic phosphonate is formed at elevated temperatures, and further low-temperature ROP is needed for the synthesis of PCPA [51]. In addition, Penczek and coll have proposed that for the successful synthesis of high-MW polymer the alcohol ROH has to be removed as fast as possible [52]. 

Relatively high-MW poly(alkylene phosphonates) (*M*_n_ = 9.3–28 kDa) were obtained by the reaction of (MeO)_2_P(O)H with HO–(CH_2_)*_n_*–OH (*n* = 5–10, 12) [23]. Polytransesterification of dimethyl phosphonate (MeO)_2_P(O)H and poly(ethylene glycol)s with *M*_n_ 200 Da (PEG200) and 600 Da (PEG600) resulted in copolymers with *M*_n_ = 3.5 and 7.1 kDa, respectively [24,25]; similar results were obtained using PEG400, transesterification was conducted within 5 h at 135 °C under atmospheric pressure, and then 4 h at 160 °C plus an additional 15 min at 185 °C in vacuo (1 Torr), degree of polymerization (*DP*_n_) was 28 [26]. The reaction of H(OCH_2_CH_2_)_13_O H with (MeO)_2_P(O)H also resulted in the formation of the polymer (*M*_n_ = 13.5 kDa) [27]. Poly(1,2-propylene glycol) (PPG)-based oligo(alkylene phosphonate)s with *DP*_n_ 12, 6 and 5 were synthesized with the use of PPG400, PPG1200 and PPG2000, respectively [28].

Triblock copolymers mPEG750-*b*-[(P(O)(H)O(CH_2_)_6_]_17_-*b*-mPEG750 and mPEG2000-*b*-[(P(O)(H)O(CH_2_)_6_]_17_-*b*-mPEG2000 were obtained by polycondensation of (MeO)_2_P(O)H with HO–(CH_2_)_6_–OH (4 h at 80 °C and then 9 h at 140 °C/1 Torr, 0.05 mol% Na to form the catalyst), followed by the reaction with mPEG (140 °C/1 Torr) [53].

**Scheme 5:**
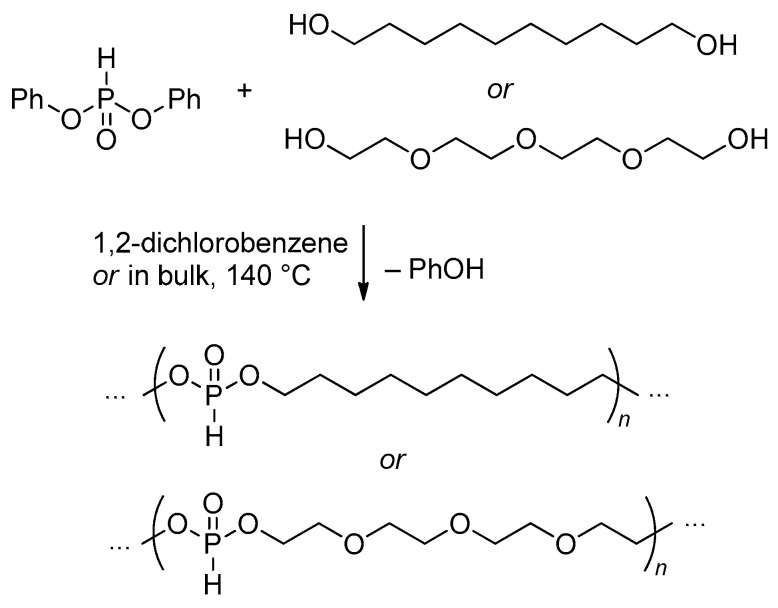
Polycondensation of diphenyl phosphonate with diols [54].

To achieve high molecular weights of the polycondensation products, Penczek and coll proposed the use of diphenyl phosphonate in reaction with diols [54]. The reaction was conducted at 140 °C with the elimination of the phenol, and PCPAs with *M*_n_ up to 40 kDa were obtained (Scheme 5).

To obtain PCPAs, PEG200- and PEG1000-based poly(alkylene phosphonate)s were oxidized by N_2_O_4_ in CH_2_Cl_2_ [24]. The same reagent was also used for the oxidation of block copolymers mPEG750-*b*-[(P(O)(H)O(CH_2_)_6_]_17_-*b*-mPEG750 and mPEG2000-*b*-[(P(O)(H)O(CH_2_)_6_]_17_-*b*-mPEG2000 in CH_2_Cl_2_ at −10 °C [53] and poly(1,2-propylene glycol)-based poly(alkylene phosphonate)s [28]. 

**Scheme 6:**
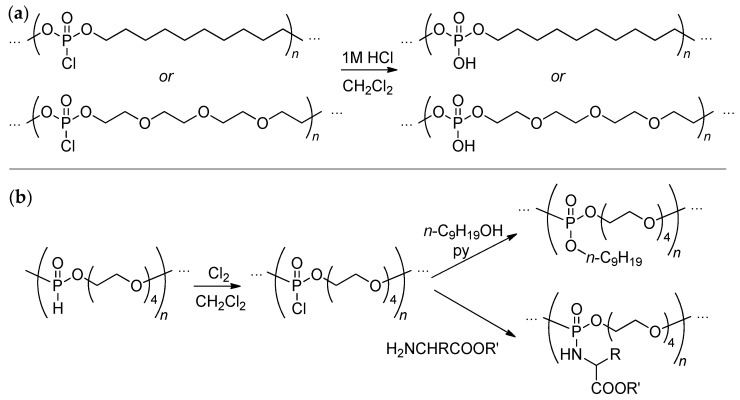
Chlorination of poly(alkylene phosphonate)s followed by the: (**a**) hydrolysis [54]; or (**b**) reaction with alcohols [23] and amino acid esters [55].

Chlorination of poly(alkylene phosphonate)s at 0 °C resulted in the formation of poly(alkylene chlorophosphate)s that can be easily hydrolyzed with a formation of PCPAs [54] (Scheme 6a) or transformed into alkoxy- [23] and amino-derivatives [55] (Scheme 6b). The degree of chlorination of poly(alkylene phosphonate)s can be varied when using trichloroisocyanuric acid as the chlorination reagent; the quantitative yield of the corresponding PCPA was confirmed by NMR monitoring of the hydrolysis of MeO[P(O)(Cl)O(CH_2_CH_2_O)_9_]_28_H in MeCN (full conversion after 15 min at 20 °C) [26]. 

**Scheme 7:**
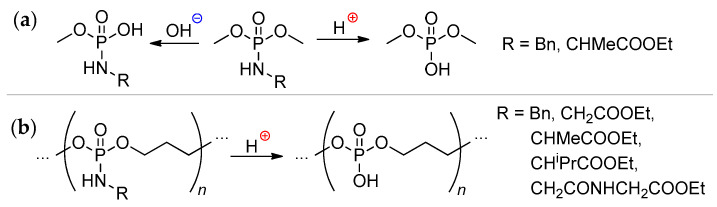
(**a**) Hydrolysis of model amidophosphates; (**b**) Acidic hydrolysis of poly(1,3-propylene amidophosphate)s [57].

Penczek and coll [56,57] have shown that the direction and selectivity of the hydrolysis of poly(alkylene amidophosphate)s depend on the pH value and the structure of the substituents in a nitrogen atom. When studying model amidophosphates, preferential cleavage of the P–O bond was detected at alkaline conditions, whereas at acidic conditions (MeO)_2_P(O)OH was the main reaction product (Scheme 7a). Poly(1,3-propylene amidophosphate)s demonstrated similar chemical behavior (Scheme 7b) except for an O-ethyl-GlyGly derivative that formed 1,3-PPPA in both acidic and alkaline conditions. At pH~8 and 37 °C the P–NH bond was hydrolyzed 3–4 times faster than the P–O bond in the main chain [56].

Another method of the transformation of poly(alkylene phosphonate)s to poly(alkylene phosphate)s uses the Atherton–Todd reaction [25]. In particular, this reaction was used in the synthesis of PCPAs containing (OCH_2_CH_2_)_13_ spacers between phosphate groups [27]. In conclusion of this section, one should refer to the successful synthesis of the polymers containing –OP(O)(H)O–(CH_2_)_x_– units (x = 10, 17, 21, 46) with *M*_n_ 11–25 kDa by the reaction of the corresponding diols with dimethyl phosphonates [58]. These polymers were not transformed to PCPAs, there was only one step to polyethylene mimicking polymers containing phosphate fragments in the main chain (note that similar polymers were nevertheless obtained by Wurm and coll. with the use of the ADMET approach, see Section 2.4).

#### 2.2.4. Polycondensation of (ω-Hydroxyalkyl)phosphonic Acids

**Scheme 8:**
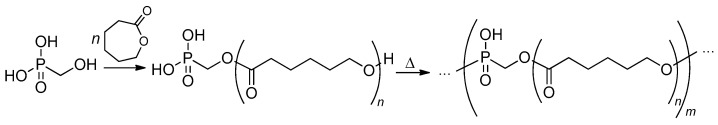
Synthesis and polycondensation of (HO)P(O)CH_2_O(εCL)_n_H [60].

In 2020, [59] Penczek and coll. have shown that hydroxymethyl phosphonic acid can act as a catalyst and initiator of the ROP of ε-caprolactone (εCL) with the formation of εCL oligomers containing reactive groups on both ends of the macromolecule. Very recently they demonstrated that these oligomers can be subjected to polycondensation at 100–110 °C with a formation of PCPAs (*M*_n_ up to 25 kDa) (Scheme 8) with mostly linear microstructure (^31^P NMR data) [60]. 

### 2.3. ROP of Cyclic Phosphorus-Containing Monomers and Post-Modification

#### 2.3.1. Synthesis of Cyclic Phosphorus-Containing Monomers

**Scheme 9:**
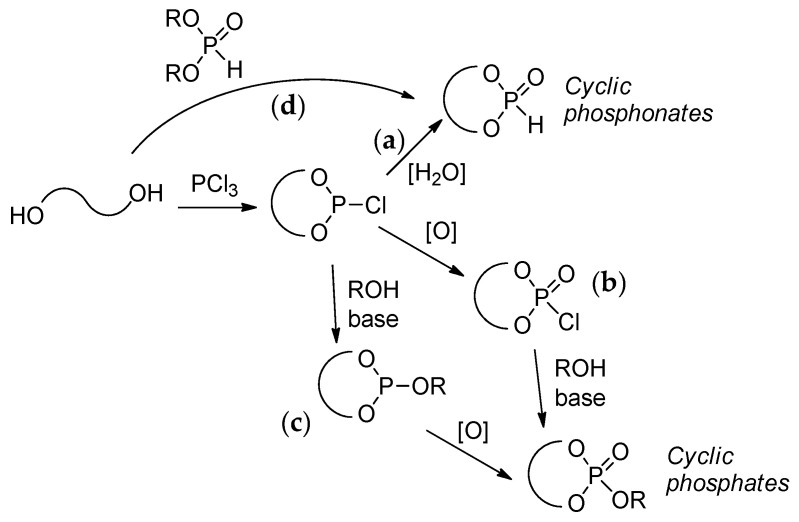
Common synthetic approaches to cyclic phosphorus-containing monomers for ROP.

The key stage of the preparation of both cyclic phosphonates and cyclic phosphates is a reaction of diols with PCl_3_ resulting in cyclic chlorophosphites [61] that can be hydrolyzed with the formation of cyclic phosphonates (Scheme 9a) or oxidized to chlorophosphates with subsequent substitution of Cl atom by alkoxy fragment that results in cyclic phosphates (Scheme 9b). In some cases, the synthesis of cyclic phosphates is based on reverse reaction sequence, i.e., substitution of Cl in chlorophosphite followed by oxidation (Scheme 9c) [62]. Cyclic phosphonates can also be synthesized by the reaction of diols with dialkyl phosphonates [63,64] (Scheme 9d).

**Scheme 10:**
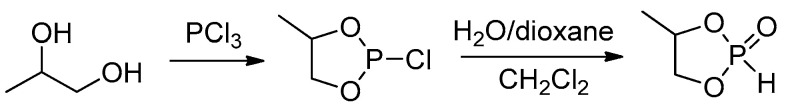
Synthesis of 4-methyl-2-oxo-2-hydro-1,3,2-dioxaphosphol [65].

Hydrolysis of chlorophosphite was carried out in CH_2_Cl_2_ solution with a mixture of water and 1,2-dioxane (Scheme 10). It was essential to use slightly less than the stoichiometric amount of water (0.8 equiv.), otherwise undesirable polymerization occurred [65].

**Scheme 11:**
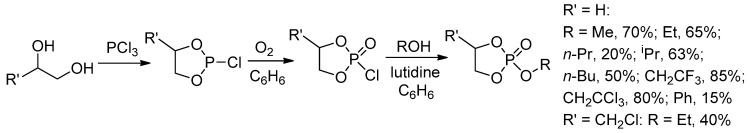
Synthesis of five-membered cyclophosphates, the yields on the last sage are given [67].

The first systematic studies of the synthesis of five-membered cyclic phosphates (2-alkoxy-2-oxo-1,3,2-dioxaphospholanes, Scheme 11), based on the reaction of cyclic chlorophosphates with ROH, were conducted by Penczek et al. back in the late 1970s [66,67]. The synthesis of 2-chloro-2-oxo-1,3,2-dioxaphospholane was optimized recently by Becker and Wurm [68]. 2-Chloro-1,3,2-dioxaphospholane was obtained with 67% isolated yield, and subsequent CoCl_2_-catalyzed oxidation by dried air resulted in the obtaining of cyclic chlorophosphate that was separated by vacuum distillation, the yield was 70%. Additionally, note that the efficient continuous flow method of the end-to-end preparation of cyclic phosphate monomers with a semi-continuous modular flow platform was developed very recently by Monbaliu and coll. [69].

2-Methoxy-2-oxo-1,3,2-dioxaphospholane (methyl ethylene phosphate, MeOEP) contained, after distillation, an impurity of (MeO)_2_P(O)OCH_2_CH_2_Cl, and the final purification involved treatment with an Na mirror. The reaction of cyclic chlorophosphates with alcohols has limitations on the substrate. Primary and secondary alcohols usually give satisfactory yields of cyclic phosphates [62,67], while *tert*-butanol does not react in the right way due to the low reactivity of *tert*-butanol at ambient conditions and low thermal stability of *^t^*BuOEP.

The choice of the base is essential in the synthesis of cyclic phosphates by the reaction of chlorophosphates with alcohols. The presence of the traces of the ammonium salts complicates the separation of cyclic phosphates because of their acid-catalyzed polymerization. The use of lutidine was proposed in the first work devoted to the synthesis of ethylene phosphates [67], and it was this base that was used in the synthesis of unstable 2-benzyloxy-2-oxo-1,3,2-dioxaphospholane (benzyl ethylene phosphate, BnOEP) [70].

**Scheme 12:**
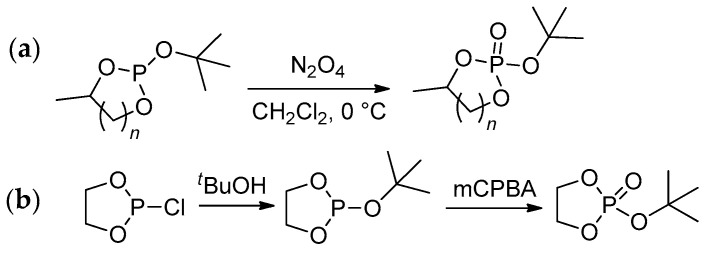
Synthesis of *tert*-butyl alkylene phosphates. (**a**) oxidation by N_2_O_4_ [71]; (**b**) oxidation by *m*PCBA [62].

Because of the low thermal stability of ^*t*^BuOEP and other *tert*-butyl alkylene phosphates, alternative approaches to these valued monomers were developed. Nakamura et al. have used oxidation of cyclic phosphites by N_2_O_4_ [71] (Scheme 12a), and recently Nifant’ev et al. proposed a two-stage approach based on reaction of 2-chloro-1,3,2-dioxaphospholane with *tert*-butanol followed by oxidation of 2-*tert*-butyl-1,3,2-dioxaphospholane by 3-chloroperbenzoic acid (*m*CPBA) [62] (Scheme 12b). 

The synthesis of deoxyribose-based five-membered cyclic phosphonate stands somewhat apart from most other 1,3,2-dioxaphospholane derivatives, this compound was obtained by the reaction of methyl-2-deoxyribofuranose with P(NEt_2_)_3_ [72].

#### 2.3.2. ROP of Cyclic Phosphorus-Containing Monomers

**Scheme 13:**
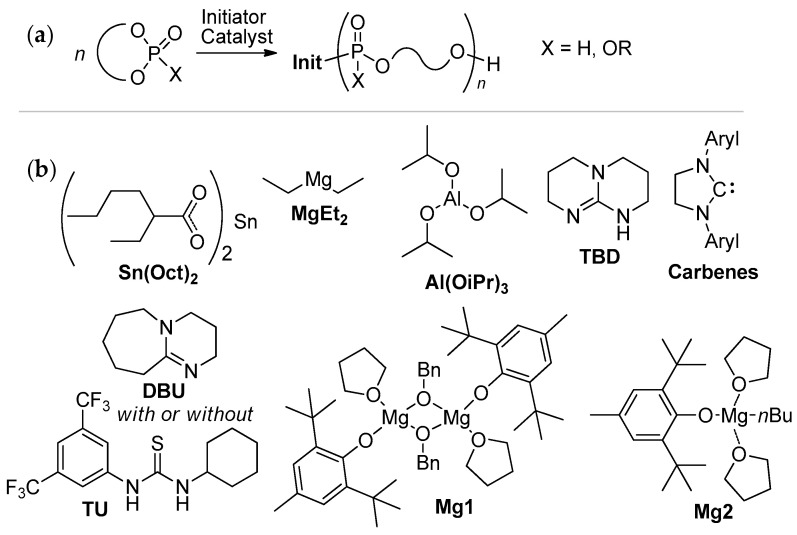
(**a**) ROP of cyclic phosphonates and phosphates; (**b**) Catalysts used in synthesis of polymers suitable for post-modification to polyphosphodiesters.

ROP of cyclic phosphonates and phosphates (Scheme 13a) represents the common strategy of the controlled synthesis of functional biodegradable polymers [30,31]. This process is subject to the general thermodynamic rules for the ROP of cyclic monomers [73] that predict high reactivity of more strained five-membered cycles [67,74,75,76] and temperature-dependent reactivity of six-membered cycles [75,76]. Different catalysts have been used successfully in controlled ROP of cyclic phosphonates and phosphates with the formation of polyphosphoesters (PPEs) (Scheme 13b). The data on the synthesis of polymers suitable for post-modification to polyphosphodiesters are summarized in Table 1.

MeOEP represents the simplest five-membered cyclic phosphate. Minimal sterical hindrance in phosphorus atoms complicate the ROP of MeOEP by the formation of branched polymers. Coordination catalyst Mg1 demonstrated high activity in polymerization of MeOEP at −20 °C with a formation of a mainly linear polymer [62,91]; however, when using an organocatalyst TBD, highly branched polymers formed [62,79]. Nifant’ev and coll. found that TBD-catalyzed polymerization of MeOEP in the presence of trimethyl phosphate leads to linear poly(MeOEP) with given *DP*_n_ and narrow molecular weight distribution (MWD) even at a >99% monomer conversion degree [79]. Note that the DBU/TU catalyst was moderately active in the polymerization of MeOEP [62,78]. Polymerization of other sterically non-hindered monomer 4-(acetoxymethyl)-2-methoxy-2-oxo-1,3,2-dioxaphospholane (Table 1, Entry 2), initiated by ^i^Bu_3_Al, was found to be a reversible process [50].

Besides MeOEP polymerization, TBD/BnOH-catalyzed ROP of –NHCH_2_CH_2_OMe-substituted ethylene phosphate with the formation of almost linear homopolymers, DBU was inactive in this reaction [90]. Poly(*^t^*BuOEP) was first obtained by Nakamura et al. back in 1981 with the use of Et_2_Mg initiator [71]. The reaction was conducted at an elevated temperature (40 °C) and took an extended period of time (10 h). The polymer of methyl-substituted analog of *^t^*BuOEP was obtained under the same conditions. In the ROP of six-membered *tert*-butyl cyclic phosphate, partially hydrolyzed El_3_Al was used as a catalyst [71]. Nifant’ev and coll. preferred to polymerize *^t^*BuOEP with the use of coordination catalyst Mg1, including the synthesis of block copolymers containing poly(*^t^*BuOEP) fragments [62,86,87,92].

In the end of this section, it should be noted that poly(phosphoester)s can be obtained by ring-opening metathesis polymerization of unsaturated cyclic phosphates [93]; however, this synthetic approach has not been applied to polyphosphodiesters. In addition, hypothetic structures of the main-chain PCPAs are not limited by ‘diesters’, and cyclic phosphonates (e.g., 2-methoxy-1,2-oxaphospholane 2-oxide [94]) might be considered as starting monomers for the synthesis of a new structural type of main-chain PCPAs using ROP and post-modification. 

#### 2.3.3. Post-Modification of the Poly(alkylene phosphonate)s

**Scheme 14:**
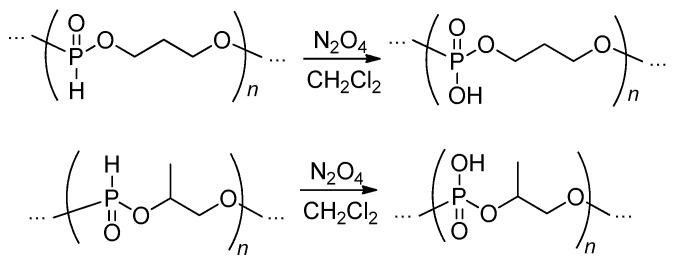
Oxidation of poly(alkylene phosphonate)s [22,50,65,76].

Oxidation of the polymers containing –P(O)H– fragments in the main chain represents a promising synthetic approach to PCPAs. In earlier studies, N_2_O_4_ in CH_2_Cl_2_ was found to be an efficient oxidizing reagent (Scheme 14), the resulting polyacids precipitated [22,50,65,76]. Wang et al. reported the use of DMF as a solvent for oxidation [95]. It is worth pointing out here that the formation of HNO_3_ during oxidation may assist the cross-linking between PCPAs’ polymer chains thereby decreasing the control on polymer MWD and architecture, thus, for example poly(1,2-propylene phosphoric acid) (1,2-PPPA) synthesized in DMF had *M*_w_ = 12.9 kDa and *Ð* = 2.6 [95].

**Scheme 15:**
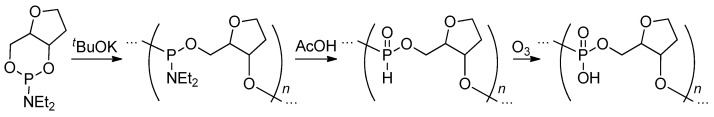
Cyclic phosphoramidite-based approach to PCPAs [72].

In an early work of Penczek’s group, the reaction with O_3_ was proposed as an efficient method of the transformation of poly(alkylene phosphonate)s to corresponding polyphosphates [72] (Scheme 15). Note that starting poly(alkylene phosphonate) was obtained via ROP of cyclic phosphoramidite followed by acid hydrolysis of the polymer obtained.

#### 2.3.4. Post-Modification of Poly(alkylene phosphate)s

**Scheme 16:**
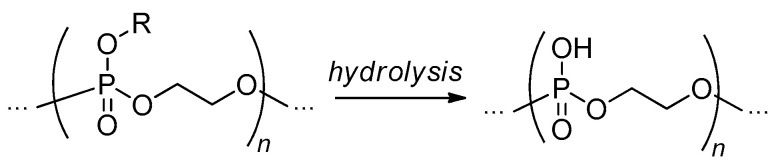
Hydrolytic pathway to PEPA.

The most evident synthetic pathway to PEPA is based on hydrolysis of the ester side groups with a maintaining of poly(alkylene phosphate) backbone (Scheme 16). The first attempt of such hydrolysis was made by Gehrmann and Vogt back in 1981 with the use of 1-oxo-2,6,7-trioxa-1-phosphabicyclo [2.2.l] heptane homopolymer of unidentified structure [96]. 

For poly(MeOEP), the dependence of the ratio of hydrolysis of the methyl ester (side group) and the backbone was established by Baran and Penczek by an example of the model linear phosphate (MeOCH_2_CH_2_O)_2_P(O)(OMe) [97], the ratio of the rate constants k_side_/k_backbone_ in water at 25 °C was ~5.0 at pH 2 and becomes equal to unity at pH ~12. Evidently, such selectivity is insufficient for the synthesis of PEPA from poly(MeOEP) with the retention of the polymer backbone.

**Figure 1:**
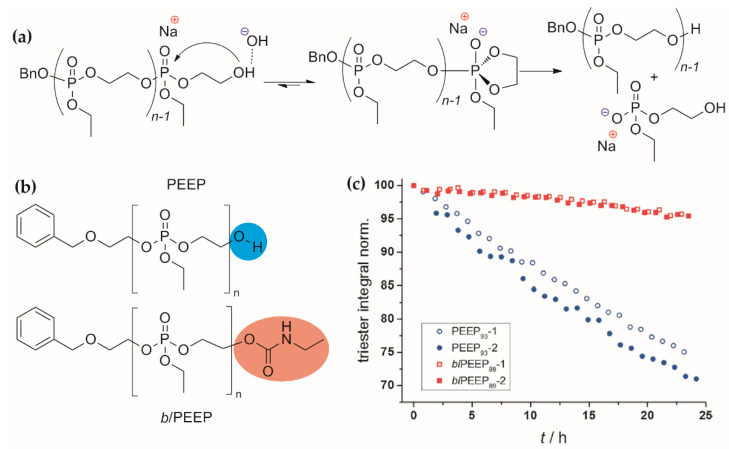
(**a**) Backbiting mechanism of hydrolytic degradation of poly(alkyl ethylene phosphate)s; (**b**) Structures of polymers with CH_2_CH_2_OH and urethane-blocked CH_2_CH_2_OH end-groups; (**c**) Degradation profile of PEEP and *bl*PEEP derived from ^31^P NMR spectra (two runs for each polymer are shown). Reprinted with permission from [78]. Copyright (2018) Elsevier B. V.

In addition, Wurm and coll. recently conducted a separate study of the hydrolysis of poly(MeOEP) and poly(EtOEP) [78] under both acidic (at pH 0, 1M HCl) and basic (pH 11, Na_2_CO_3_/NaOH buffer) conditions. They found that under basic conditions these polymers undergo a backbiting hydrolysis resulting in the release of alkyl (2-hydroxyethyl) hydrogen phosphate as the main degradation product (Figure 1a). High hydrolytic stability of polymer with urethane-blocked CH_2_CH_2_OH end-group (Figure 1b,c) confirms this mechanism. In this way, the hydrolytic approach to PEPA should not be overestimated. That is probably why the search for other nucleophilic agents and leaving groups were carried out to develop efficient synthetic approaches to PEPA and other poly(phosphodiesters) based on poly(alkylene phosphate)s.

Already in the first communication on coordination ROP of MeOEP, Penczek demonstrated high efficiency of the use of aq. Me_3_N in the synthesis of PEPA (~90% dealkylation efficiency) [22]. The reaction of poly(MeOEP) (*M*_n_ = 22 kDa) with 30% aq. Me_3_N at 50 °C for 10 h, followed by a pass through a cation exchange resin to exchange the NMe_4_^+^ ions by protons resulted in high-MW PEPA with 85% yield [98]. A similar approach was used by Iwasaki group in the preparation of PEPA, cholesterol-(PEPA)*_n_* (*n* = 24, 46, 106) and different PEPA-containing copolymers [80,81,82,99,100,101]. A sufficiently high selectivity was achieved when Et_3_N was used as a dealkylation agent for the linear high-MW poly(MeOEP): the rate of dealkylation of the side groups and the backbone was ~500:1 [22].

Dealkylation of the polymer obtained by ROP of 4-CH_2_OAc substituted MeOEP (Table 1, Entry 2) was performed by using aq. R_3_N or NaI in acetone solution. The best results were obtained by the latter method. However, the extent of dealkylation did not exceed 80% [50].

To obtain PEPA, Wooley and coll. Conducted hydrolysis of poly(ethylene phosphoramidate) obtained by ROP of the corresponding cyclic substrate (Scheme 16, R = –NHCH_2_CH_2_Ome) in three different acidic buffer solutions having pH values of 1.0, 3.0 and 5.0 [90]. At pH 5.0, only 7% of the phosphoramidate bonds were converted into phosphate in 130 h. At pH 3.0, greater than 23% of the phosphoramidate bonds were cleaved over 130 h. At pH 1.0, complete hydrolysis was reached within 10 h. Significantly faster and selective formation of PEPA was observed when polymer of allyl ethylene phosphate (Scheme 16, R = –CH_2_CH=CH_2_) was treated by PhSNa in DMF/H_2_O [84,85]. Additionally, note that partial (~20%) hydrolysis of the homopolymer of but-3-yn-1-substituted ethylene phosphate (for structural formula see Table 1, Entry 7) occurred during thiol−yne click reaction with (*L*)-cysteine [102].

**Scheme 17:**
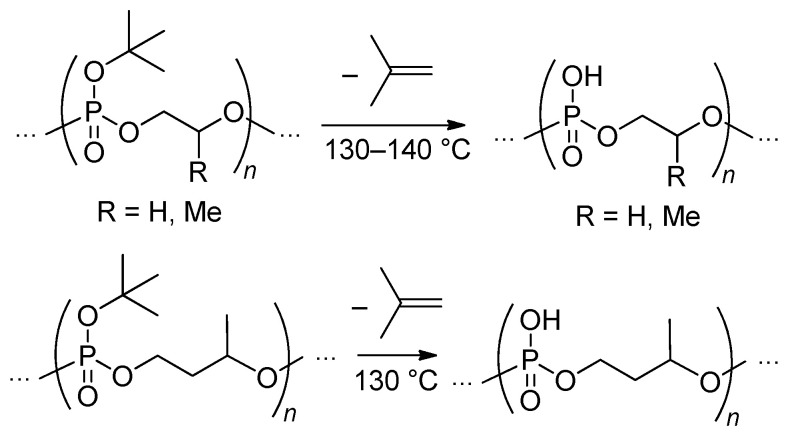
Formation of PEPA, 1,2-PPPA and poly((1,3-bulylene)phosphoric acid) [71].

Another efficient way to PCPA is based on thermolysis of polyphosphates containing *tert*-butoxy fragments. Even at 1981 Nakamura and coll. have shown formation of the corresponding PCPAs with elimination of isobutylene during thermolysis of poly(*^t^*BuOEP) at 140 °C, as well as poly(4-methyl-2-hydroxy-1,3,2-dioxaphospholane 2-oxide) and poly(4-methyl-2-hydroxy-1,3,2-dioxaphosphorinane 2-oxide) at 130 °C (Scheme 17) [71]. The authors have noted that copolymers were partially cross-linked due to formation of P–O–P bonds under heat.

**Figure 2:**
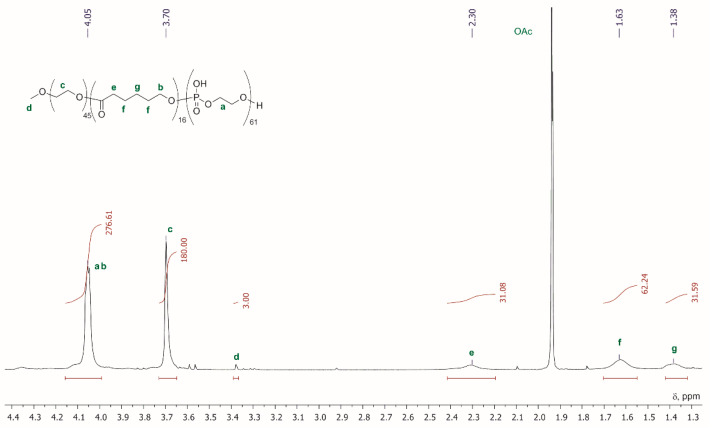
^1^H NMR spectrum (400MHz, D_2_O, 20 °C) of PEPA-containing triblock copolymer obtained after thermolysis of mPEG_2000_-*b*-(εCL)_16_-*b*-(*^t^*BuOEP)_61_H in D_2_O at 80 °C in the presence of NaOAc. Reprinted with permission from [86]. Copyright (2018) Elsevier B. V.

To avoid similar cross-linking, Nifant’ev and coll. proposed the use of proton solvents (water, MeOH) for thermolysis of poly(*^t^*BuOEP) [86]. Due to the presence of proton solvents, the reactions were completed after 15 min (in H_2_O) or after 1 h (in MeOH) at 80 °C. By this method, copolymers containing poly(*^t^*BuOEP) blocks were successfully converted into PEPA-containing macromolecules (Figure 2). The presence of bases (NaOAc, Na_2_CO_3_) completely blocked P–O–P cross-linking [86].

**Scheme 18:**
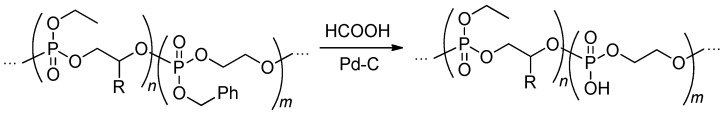
The synthesis of PEPA copolymers based on poly(BnOEP) [70,88,89].

Another common approach to PCPAs is based on the lability of benzyl phosphates towards catalytic hydrogenolysis. To avoid the use of H_2_, Iwasaki et al. carried out elimination of the BnO groups in copolymers poly(EtOEP)-*ran*-poly(BnOEP) via 4 h of stirring in HCOOH in the presence of Pd/C (8 wt%) [70,88,89] (Scheme 18), note that in [89] cholesterol was used as a ROP initiator.

In the end of this Section, it would be worth highlighting that the use of ROP in controlled synthesis of PCPAs is still limited by the next significant drawbacks:

- Loss of control over polymer architecture and MWD: sterically non-hindered cyclic phosphates can form highly branched poly(alkylene phosphate)s. Switching between the ‘living’ (linear polymer, *Đ*_M_~1) and ‘immortal’ (transesterification of the polymer chain, branched polymer, *Đ*_M_ > 1) ROP modes can occur at elevated temperatures and/or in case of wrong catalyst’ choice. Moreover, even in the presence of ‘good’ catalysts, complete conversion of the monomer greatly increases the risk of subsequent transesterification.
- This is why better chain control can be achieved when using sterically hindered cyclic phosphates, e.g., *^t^*BuOEP, despite its minor synthetic accessibility and very low reactivity that limits the use of this monomer in the synthesis of stat- and block-copolymers.
- The use of cyclic phosphonates eliminates the problem of branching and *DP*_n_ control, but severe oxidation of the P–H bond at the final stage puts the end to a convenient option to introduce biomolecules or usable functional groups at the stages of ROP initiation or termination.
- The nature of the catalytic ROP imposes severe restrictions on the nature of the side substituent R in the molecule of cyclic phosphate (Scheme 16). So, for example, the –CH_2_CH_2_CN group, widely used in *automated* (!) synthesis of DNA analogs [103] and in synthesis of PCPAs with the use of ring-opening metathesis polymerization (ROMP) [104], has not found application in the ROP/deprotection approach to PCPAs, despite the fact that the synthesis of six-membered cyclic phosphate with this substituent was synthesized by Lapienis and Penczek back in 1977 [66].
- Additionally, in general, between fundamental studies of the ROP/deprotection approach to PCPAs in the late 1970s–1980s (conducted for the most part by the Penczek’ group) and relatively recent works (scientific groups of Wooley, Wurm, Iwasaki, Nifant’ev), a two-decades gap in investigations is clearly visible, which affected the progress in this scientific direction.

### 2.4. Metathesis Polycondensation

**Scheme 19:**
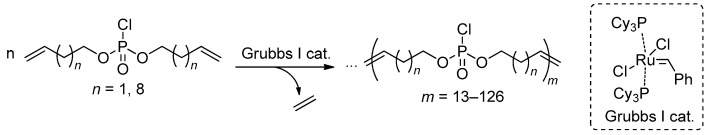
The synthesis of chlorophosphate polymers with the use of ADMET polycondensation [32].

In 2014 Wurm and coll. proposed an efficient synthetic approach to polyphosphodiesters based on ADMET polycondensation of bis(alkenyl) chlorophosphates, catalyzed by the first generation Grubbs catalyst [32]. In bulk polymerization, *DP*_n_ of 39 was achieved, and when using 1-chloronaphthalene as a solvent, *DP*_n_ was 47 and 126 for ‘chloro monomers’ containing –(CH_2_)_2_– and –(CH_2_)_9_– spacers between vinyl and phosphate fragments, respectively (Scheme 19). Functionalized PCPAs were then obtained by the reactions of poly(alkylidene chlorophosphate)s with PhOK or (2-hydroxyethyl)methacrylate (HEMA) in the presence of water.

**Scheme 20:**
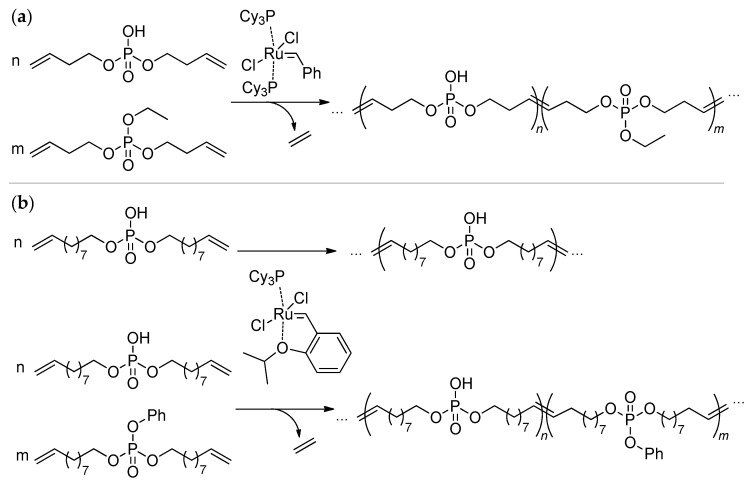
The synthesis of PCPAs with the use of ADMET (co)polycondensation of bis(alkenyl) phosphates with short (**a**) [33] and long (**b**) [34] hydrocarbon fragments.

During further studies, copolymers containing P–OH and P–OEt substituents (Scheme 20a) in 2:8 and 1:9 ratios (*M*_n_ = 19.3 and 10.3 kDa, respectively) and low-MW homopolymer of (CH_2_=CHCH_2_CH_2_O)_2_P(O)OH (*M*_n_ = 1.7 kDa) were obtained [33]. The reaction was also conducted in the presence of the first-generation Grubbs catalyst, the *M*_n_ of the 1:4 copolymer was 19.3 kDa. To prepare potentially biodegradable analogs of polyolefins, Wurm and coll. [34] also used ADMET polycondensation of HO–P(O)(O(CH_2_)_8_)CH=CH_2_)_2_ and copolycondensation of this monomer with PhO–P(O)(O(CH_2_)_8_)CH=CH_2_)_2_ in different ratios in the presence of Hoveyda−Grubbs catalyst (Scheme 20b). After catalytic hydrogenation, homopolymers demonstrated promising physico-mechanical characteristics.

**Scheme 21:**
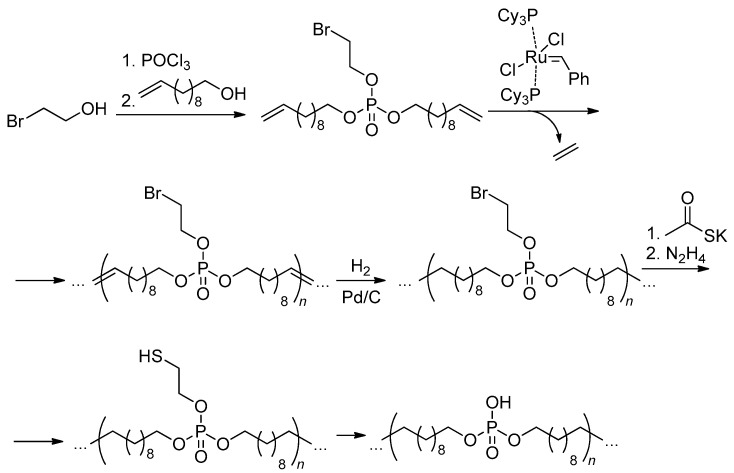
The synthesis of ‘polyethylenes’ containing –P(O)OH– fragments [36].

The use of monomers containing highly reactive P–Cl and P–OH bonds can complicate ADMET polycondensation. Wurm and coll. demonstrated feasibility of the 2-acetylthioethyl ester fragment as a protective group for the P–OH functionality in low molecular weight phosphates as well as polyphosphates [35]. In order to obtain ‘polyethylenes’ containing –P(O)OH– fragments and –(CH_2_)_20_– spacers between them, Wurm and coll. [36] synthesized a new monomer containing –OCH_2_CH_2_Br substituent at phosphorus atom. ADMET polycondensation and subsequent hydrogenation resulted in poly(phosphotriester), its deprotection to PCPA was carried out in two stages using 2-acetylthioethyl ester protective group (Scheme 21)

### 2.5. Other Synthetic Approaches to Polyphosphodiesters

#### 2.5.1. The Use of Unsaturated 2-Cyanoethyl Phosphates

**Scheme 22:**
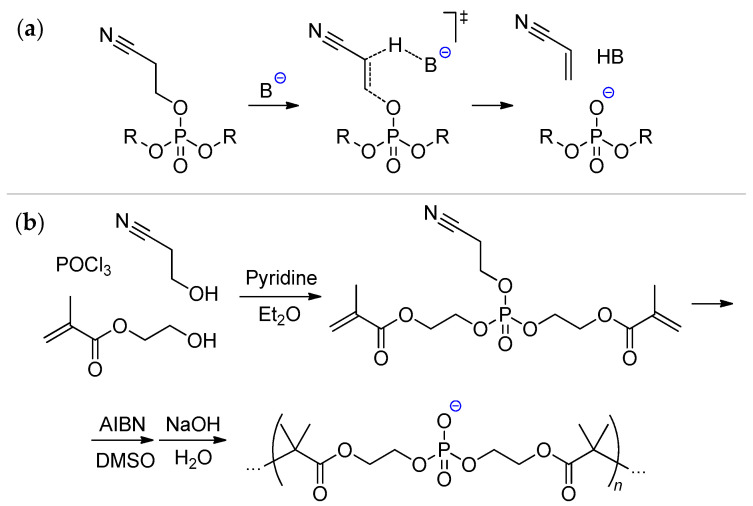
(**a**) Base-induced transformation of (2-cycnoethyl)phosphates to phosphodiesters; (**b**) Synthesis of bis(methacryloyl)(2-cycnoethyl) phosphates and PCPAs [104].

The synthesis of phosphodiester hydrogels (this Section) and sequence-defined oligophosphodiesters (see Section 2.6) relies on the use of sensitivity of 2-cyanoethyl phosphates to bases (Scheme 22a). So, for example, bis(methacryloyl)(2-cyanoethyl)phosphate was synthesized, polymerized, and deprotected with a formation of PCPAs (Scheme 22b) [104].

#### 2.5.2. Bis(methacrylate) Phosphonates and Their Post-Modification

**Scheme 23:**
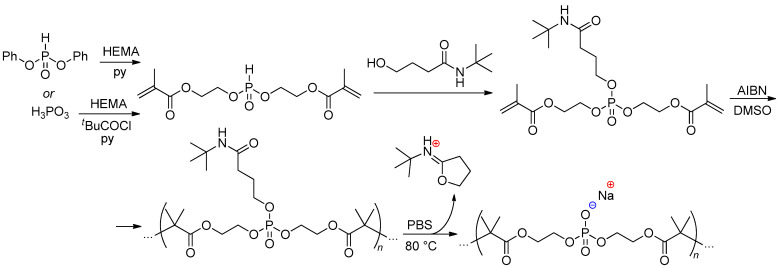
Synthesis of PCPAs based on bis(methacryloyl)phosphonates [105].

Diliën and coll. proposed efficient synthetic approach to monomers for the synthesis of PCPA-containing hydrogels, based on the reaction of (PhO)_2_O(O)H or H_3_PO_3_ with 2-hydroxyethylmethacrylate (HEMA), followed by the Atherton–Todd reaction with N-*tert*-butyl-4-hydroxybutanamide and CCl_3_Br/NEt_3_ (Scheme 23). Free-radical polymerization of this monomer followed by thermal deprotection via elimination of stable five-membered iminoester resulted in formation of the polymers containing main-chain –P(O)OH– fragments [105].

#### 2.5.3. Hydrolytic Polymerization of Spiro(acylpentaoxy)phosphoranes

**Scheme 24:**
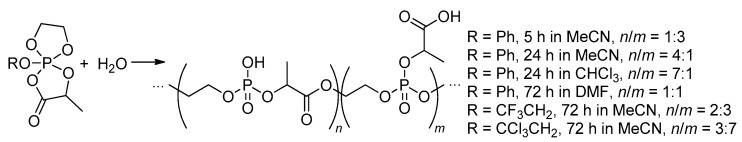
Preparation of PCPAs via thiol-ene polyaddition and subsequent oxidation by hydrogen peroxide [106].

Saegusa and coll. have demonstrated that spiro-phosporanes can react with water to form polymers containing phosphodiester and phosphotriester monomer units [106]. The ratio of monomer units was determined by the reaction time and the solvent (Scheme 24), the maximum MW was 2.3 kDa. 

#### 2.5.4. Thiol-Ene Polyaddition

**Scheme 25:**
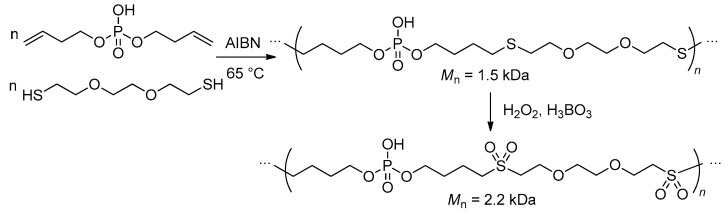
Preparation of PCPAs via thiol-ene polyaddition and subsequent oxidation by hydrogen peroxide [107].

Recently Wurm and coll. proposed a new approach to PCPAs based on metal-free-radical thiol-ene polyaddition of dithiol comonomer and bis(alkenyl) phosphate to produce alternating copolymer with hydrophilic ethylene glycol segments in the polymer backbone (Scheme 25). To increase the hydrophilicity of the polymer, it was oxidized to the sulfone [107].

#### 2.5.5. Chain-End Vinyl Functionalization

**Scheme 26:**
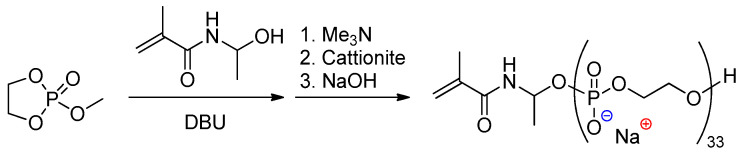
Synthetic scheme of the polyphosphoester macromonomers [101].

Iwasaki described the use of methacrylamide-containing initiator in ROP of MeOEP, followed by the reaction with Me_3_N, to obtain functionalized Na-PEP (Scheme 26) suitable for free-radical graft polymerization [101]. Strictly speaking, the products of the latter reaction cannot easily be classified as ‘main’- or ‘side’-chain PCPAs, such attribution depends on the length of the grafted polymer.

#### 2.5.6. The Use of Bridged Cyclic Phosphates

**Scheme 27:**
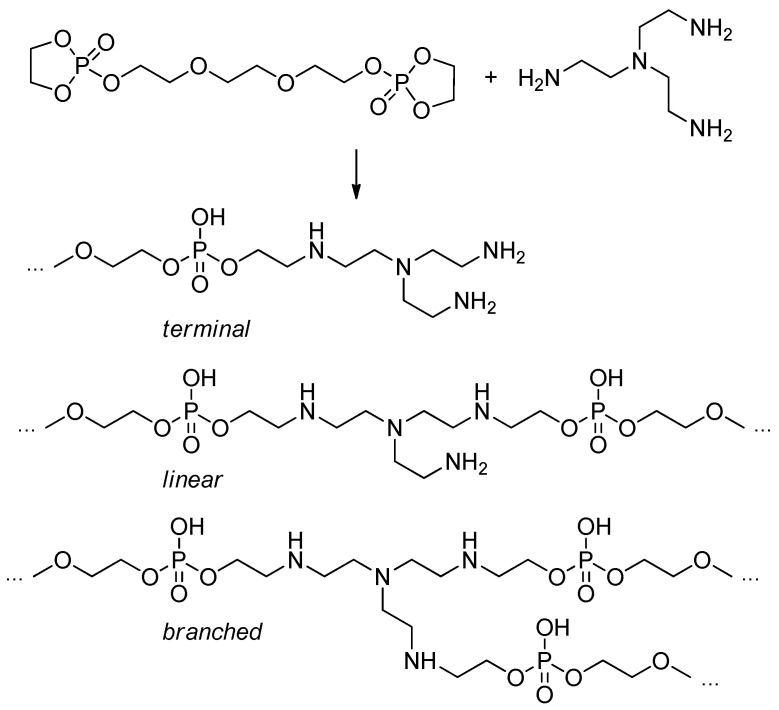
Various structures of the reaction products of 3,6-dioxaoctan-1,8-diyl bis(ethylene phosphate) with tris(2-aminoethyl)amine [108].

Highly branched phosphate nanogels were obtained by polymerization of bridged cyclic phosphoester, 3,6-dioxaoctan-1,8-diyl bis(ethylene phosphate) and tris(2-aminoethyl)amine, in the presence of Triton X-100 in cyclohexane [108]. The product of this reaction contained three types of structural fragments (Scheme 27).

#### 2.5.7. Post-Modification of Polyphosphodiesters

Polyphosphodiesters contain reactive acidic P–OH fragments and can of course be chemically modified. The reaction of PCPAs with oxirans (oxyethylation) stops when all of the acidic groups are consumed [23], the synthesis of PEGylated polyphosphoesters requires the addition of an ‘external’ acid. Iwasaki synthesized polyphosphoester containing P–OCH_2_OAc and P–OH groups by the reaction of poly(EtOEP)-*ran*-PEPA with acetoxymethyl bromide [70].

### 2.6. Sequence-Defined Oligophosphodiesters

**Scheme 28:**
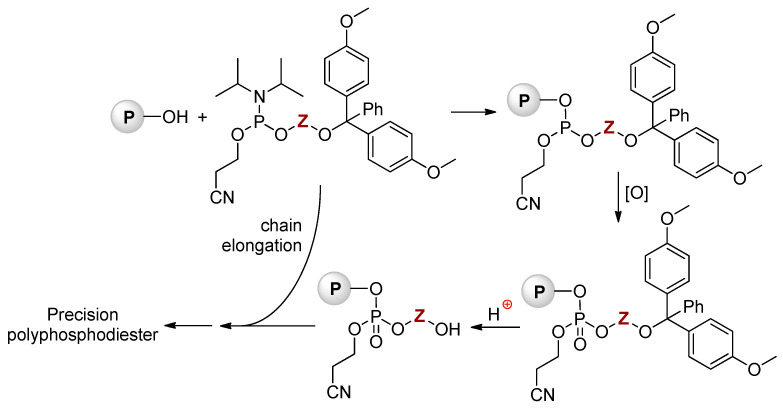
Synthesis of sequence-defined PCPAs based on ‘phosphoramidite’ chemistry [109,110].

Nucleic acids are PCPAs that serve as the primary information-carrying molecules in cells. These natural PCPAs can be considered as sequence-defined poly(phosphodiesters) containing limited numbers of the ‘building blocks’. The maximum of the researchers’ interests in this area was highest during the 1980s, organochemical approaches to artificial DNA and close DNA analogs had been reviewed by Caruthers in 1991 [103]. The synthesis of ‘artificial’ nucleic acids is based on ‘phosphoramidite’ chemistry (Scheme 28), initially developed for solid-phase DNA synthesis [103].

**Scheme 29:**
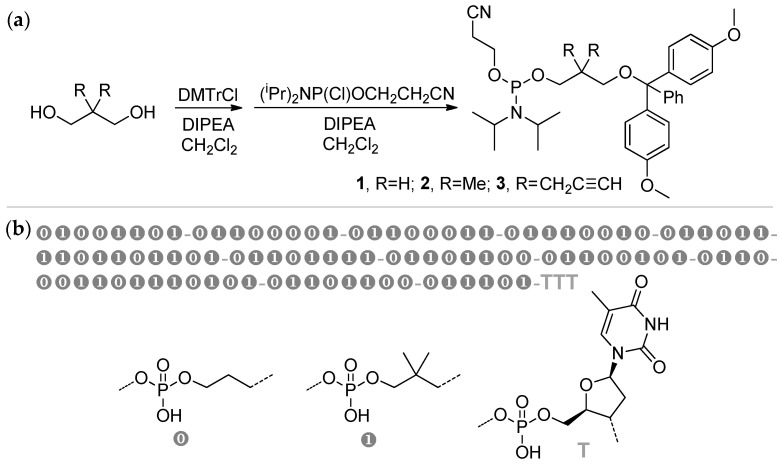
(**a**) Synthesis of the phosphoramidite monomers [113]. (**b**) The sequence-defined copolymer with *DP*_n_ > 100 and the structures of the comonomer units [109].

Sequence-defined PCPAs were recently reviewed by Häner et al. [110], Charles and Lutz [111], and by Grass et al. [112]. High efficiency of the phosphoramidite approach was demonstrated mainly by Lutz and coll. in the preparation of sequence-defined PCPAs of different structures [109,111,113,114,115]. In particular, a series of sequence-defined poly(phosphodiester)s were synthesized based on a cross-linked polystyrene bead with the use of three monomers **0**–**2** (Scheme 29a) prepared from the corresponding 1,3-diols [113]. Monomers **0** and **1** were also used in the synthesis of ‘coded’ copolymers containing deprotected comonomer units τ and υ (Scheme 29b) with a primer sequence containing three thymine nucleotides (**TTT**) [109]. During the latter study, copolymer with *DP*_n_ >100 was prepared.

In further research of ‘informational’ PCPAs, different types of spacers between phosphate fragments were investigated, including variably alkyl-substituted [116], N-(alkyl)-N,N-bis(alkylene)amine [114], N-(amidoalkyl)-N,N-bis(alkylene)amine [117], alkoxyamine [118], photo-editable substituted aryl [119]. 

**Scheme 30:**
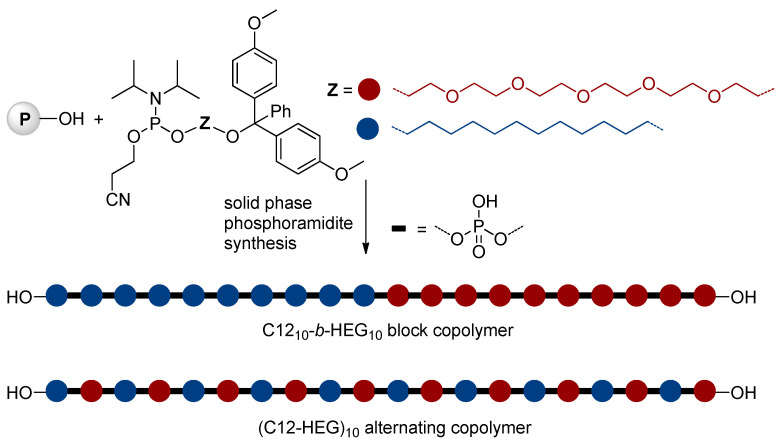
Synthesis of sequence-isomeric poly(phosphodiester)s [120].

A carefully developed strategy of the synthesis of ‘artificial’ NAs was further used in the preparation of aptamer-*b*-poly(phosphodiester) conjugates containing conventional nucleic acid fragments [115]. Interesting examples of the use of phopshoramidite chemistry was reported by Serpell et al. who have synthesized two sequence-isomeric polymers from dodecane diol (C12) and hexa(ethylene glycol) (HEG)-containing substrates, namely, C12_10_-*b*-HEG_10_ block and (C12–HEG)_10_ alternating copolymers [120] (Scheme 30).

**Figure 3:**
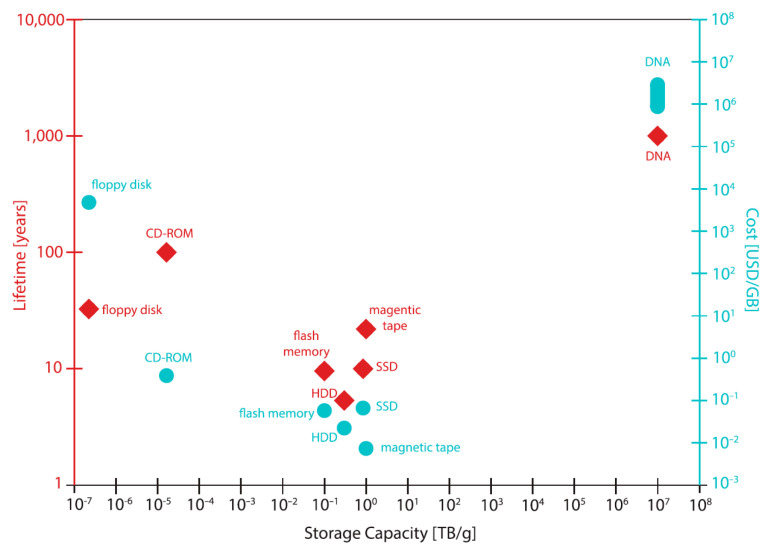
Lifetime, current storage capacity, and costs of various storage systems. Reprinted with permission from [112]. Copyright (2021) Springer Nature.

‘Reading’ of the information is no less important and a no less time-consuming task in comparison with ‘recording’ using the phosphoramidite approach [121,122]. Real prospects of the use of ‘digital’ synthetic PCPAs for data storage are unclear at the moment; however, DNA and its synthetic analogs are clear leaders among other data storage materials by the criteria of the lifetime and storage capacity. Although, this leadership is in place by the criterion of the price too (Figure 3) [112].

**Figure 4:**
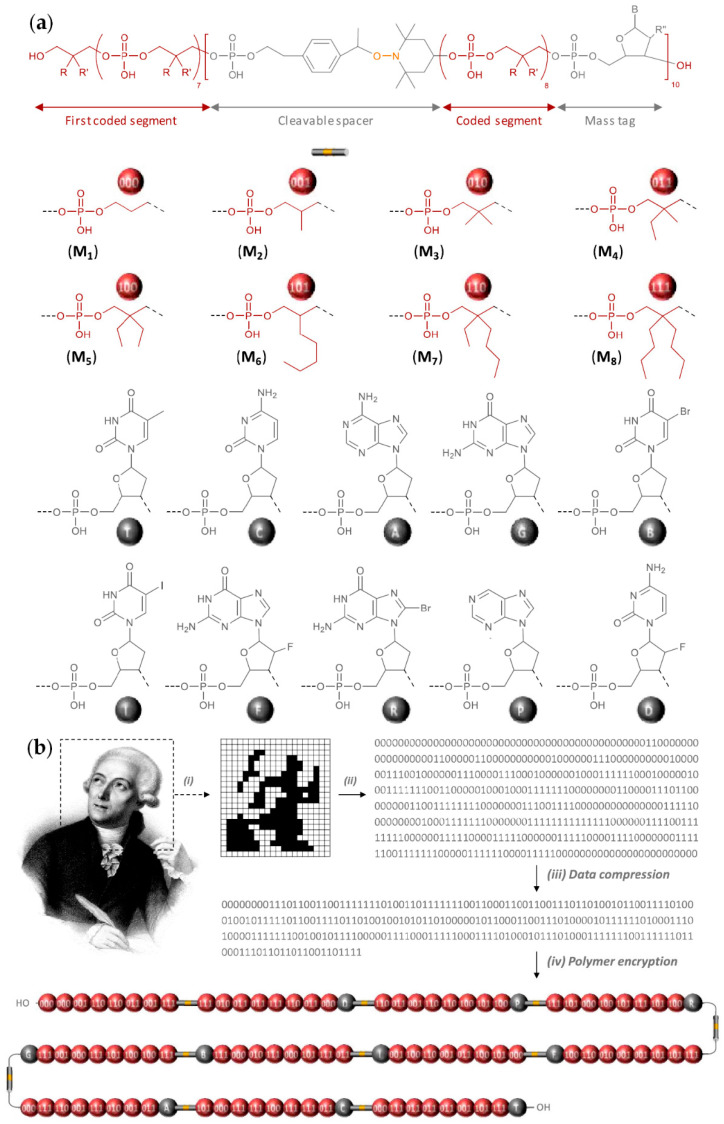
(**a**) Design of the digital polymer. General molecular structure of poly(phosphodiester) [123], structure of the eight synthons that permit to code binary information in the chains, and structure of the ten mass tags, which facilitate the decryption of the digital sequence by mass spectrometry; (**b**) Polymer encryption. (i) Pixelation of the portrait of Lavoisier (20 × 22), (ii) transformation into a 440-bits string with 0 (white) and 1 (black), (iii) compression, (iv) translation into a chemical monomer sequence employing the building blocks shown in (**a**). Reprinted with permission from [123]. Copyright (2021) Institut de France Academie des Sciences.

Lutz and coll. have vividly illustrated the efficiency of the ‘molecular’ encryption with the use of ‘digital’ PCPAs by an example of the portrait of Antoine Laurent de Lavoisier (Figure 4) [123]. 

## 3. Properties and Applications of Polyphosphodiesters 

### 3.1. Physico-Chemical Characteristics of Polyphosphodiesters

#### 3.1.1. Physical State and Mechanical Properties of Polyphosphodiesters

**Figure 5:**
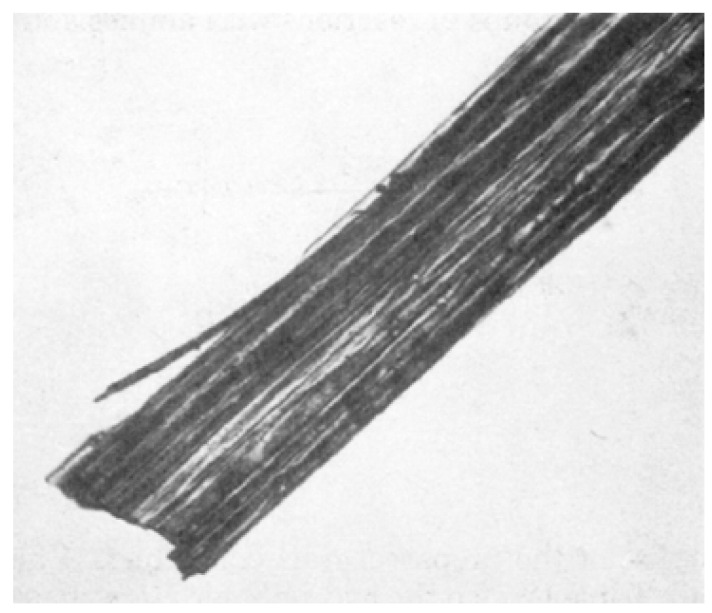
Oriented crystalline structure formed when the poly(decamethylene phosphate) sample was drawn from the melt. Reprinted with permission from [23]. Copyright (1993) American Chemical Society.

PEPA and its close analogs represent amorphous compounds, but with the increasing of the number *n* of –(CH_2_)*n*– fragments between phosphate groups beginning with *n* = 5 poly(alkylene phosphate)s demonstrate explicit crystalline behavior (Figure 5) [23].

The products of ADMET (co)polycondensation of HO–P(O)(O(CH_2_)_8_)CH=CH_2_)_2_ and PhO–P(O)(O(CH_2_)_8_)CH=CH_2_)_2_ (Scheme 20b) represent low-crystalline materials. Their hydrogenation resulted in an increase in crystallinity; however, copolymers with low content of P–OH fragments were too brittle. The increase in the supramolecular P–OH^…^O=P cross-linking as a result of an increase in the content of phosphodiester fragments showed a significant impact on the material properties: higher glass-transition and melting temperatures were observed and an increase in the storage modulus was detected. Hydrogenated homopolymer of the phosphodiester monomer also demonstrated the shape memory effect [34].

The polymer platelets were prepared by solution crystallization of polymers containing –(CH_2_)_10_– spacer between –OP(O)(OCH_2_CH_2_Br)O– fragments (*M*_n_ = 15.9 kDa, *Ð*_M_ = 1.67) [36], a pseudohexagonal crystal structure with the phosphate groups remanating from the two opposing surfaces of the crystal formed. Further surface modifications (see Scheme 21) resulted in the formation of the OP(O)(OH)O– fragments. Wurm and coll. proposed that similar PE-like polymers can be used as a general platform to design chemically functional anisotropic materials with the possibility of degradation of the phosphoester bonds combined with the crystallinity of PE. 

#### 3.1.2. Solution and Colloidal Behavior

Solutions of 1,2-PPPA in distilled water did not exhibit phase transition temperature at any concentration [95]. 1,3-PPPA demonstrates similar behavior; however, poly(1,5-pentylene phosphoric acid) swells slowly in water and forms a gel-like material after absorbing up to 1000% of H_2_O [23].

**Figure 6:**
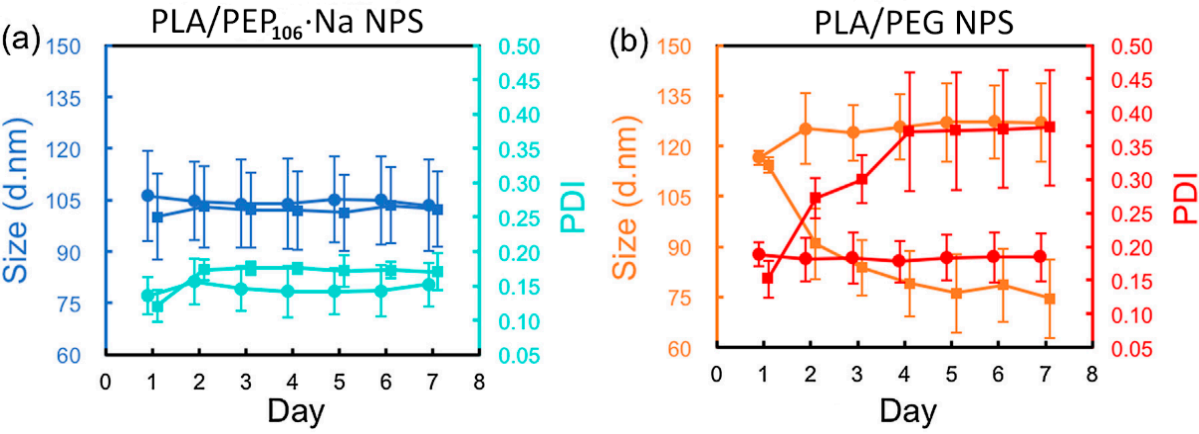
Particle size and polydispersity index (PDI) of PLA/PEP_106_·Na NPs (**a**) and PLA/PEG NPs (**b**) suspended in water (•) and PBS (▪) during days 1–7. Reprinted with permission from [80]. Copyright (2017) Elsevier B. V.

Hirano and Iwasaki have demonstrated the ability of Chol-PEPA sodium salts to form stable nano-sized micelles in combination with PLA using a solvent evaporation method for micelle preparation: when compared with Chol-PEG, PEPA derivatives have shown weak dependence of the particle size on the pH values (Figure 6) [80].

Cholesterol-containing random PEPA/poly(EtOEP) copolymers (Scheme 18) were modified to 1,2-dioleoyl-*sn*-glycero-3-phosphocholine (DOPC) vesicles. The ζ-potential of the vesicles was decreased by an immobilization copolymer, the release rate of 5-carboxyfluorescein from the vesicles containing 3% of copolymer was most reduced. In addition, the enzymatic degradation of DOPC was reduced by immobilization with the polyphosphoester ionomers [89].

A complex of cholesterol (Ch)-terminated Na-PEP with bovine serum albumin (BSA) was formed at 90 °C through the hydrophobic interactions between the lipophilic moieties of the protein and the cholesteryl groups of the Ch-Na-PEP chains. The complexes dispersed in an aqueous medium (27–51 nm, DLS data) exhibited a high stability in size for up to 1 month and efficiently inhibited the thermal aggregation and sedimentation of BSA, in contrast with Na-PEP and Ch-PEG. In addition, Ch-Na-PEP was able to protect the complexed BSA against proteolytic digestion [81].

#### 3.1.3. Chemical Stability of Polyphosphodiesters

As shown by Baran and Penczek [97], dialkyl phosphates are relatively stable in broad pH intervals. Iwasaki et al. have shown that the presence of –OP(O)(OH)O– fragments in the main chain of the poly (ethylene phosphate) copolymer containing –OEt and –OCH_2_OC(O)Me fragments significantly affects the cleavage of the latter fragments in contact with esterase and further phase behavior with a formation of thermoresponsive PPEs [70]. 

### 3.2. Metal Complexation of the Polyphosphodiesters and Polymer-Inorganic Hybrids

#### 3.2.1. Complexation of Polyphosphodiesters with Metal Ions 

Back in 1990, Wódzki and Kłosiński studied the complexation and transport of Mg^2+^ and Ca^2+^ ions by polyphosphodiesters and found that the affinity of phosphate groups for magnesium ions is strongly dependent on the type of phosphodiester linkage: the shorter spacer between phosphate groups (e.g., –CH_2_CH(CH_2_OH)–) diminished the Mg^2+^ transport and favored the Ca^2+^ transport, which had not occurred when using 1,3-PPPA [124].

Addition of Ca^2+^ to the aqueous solutions of 1,2-PPPA significantly changed the phase transition properties. At 20 °C, up to 20 wt % 1,2-PPPA solutions remained a liquid in the presence of up to 0.7 M CaCl_2_. A 25 wt % 1,2-PPPA solution was obtained at 20 °C in 0.5 M CaCl_2_, and exhibited a rapid phase transition to a nonflowing gel when the temperature was raised to 36 °C. At concentrations of 1,2-PPPA below 10 wt %, only precipitates were observed even at high temperatures. Increasing the polymer or the Ca^2+^ concentration led to lower phase transition temperatures [95].

Bis(2-hydroxyethyl)phthalate-based copolymers (Scheme 4) have demonstrated high affinity to Ca^2+^ ions, which was manifested in changes in thermal properties of copolymers (increase of the glass transition temperature and melting range, up to 15 and 30 °C, respectively) and in mechanical characteristics of the polymer films (two- to three-fold increase in Young’s modulus and hardness) [49]. However, no follow-up was given to these materials in the development of polymer scaffolds for biomedical applications.

PPG-based oligo(alkylene phosphate)s were applied as cation-selective macroionophores in a multimembrane hybrid system [28]. Their solutions in dichloroethane formed the flowing liquid membrane (FLM) circulating between two polymer cation exchange membranes, and subsequently, between two polymer-made pervaporation membranes. It was found that the copolymer macroionophores activate the preferential transport of Zn^2^+ cations from aqueous solutions containing competing Cu^2+^, Ca^2+^, Mg^2+^, K^+^, and Na^+^ cations. Depending on the MW of PPG used in the synthesis of copolymer, the following separation orders were observed: for MW 400 and 1200 Da Zn^2^+ > Cu^2+^ >> Ca^2+^, Mg^2+^, K^+^, Na^+^; for MW 2000 Da Zn^2+^ > Cu^2+^, Ca^2+^ >> Mg^2+^, K^+^, Na^+^. Due to moderate separation selectivity, complexity of the multimembrane hybrid system setup, and the unpredictable effect of additional factors (e.g., water transfer or uptake), the idea of the use of polyphosphodiesters in liquid membranes stalled.

When studying two sequence-isomeric C12_10_-*b*-HEG_10_ and (C12–HEG)_10_ copolymers, synthesized with the use of solid phase phosphoramidite approach (Scheme 30) [120], Serpell and coll. Hypothesized that in the presence of Mg^2+^ C12_10_-*b*-HEG_10_ would give conventional spherical star micelles, whereas the alternating (C12–HEG)_10_ would show no self-assembly. However, experiments with the use of Mg^2+^ containing buffer have demonstrated the formation of non-isotropic particles (107 ± 5 nm) at 4 μM concentration of (C12–HEG)_10_, and formation of large, highly anisotropic, higher order structures at 7–100 μM concentrations. It is quite possible that the detection of untypical colloidal behavior can be attributed to the use of sequence-defined copolymers, because in the case of statistical copolymers the manifestation of the similar effects is mitigated by heterogeneity of the microstructure and broadening of the MWD.

#### 3.2.2. Effects of the Polyphosphodiesters on Crystal Growth and Morphology

**Figure 7:**
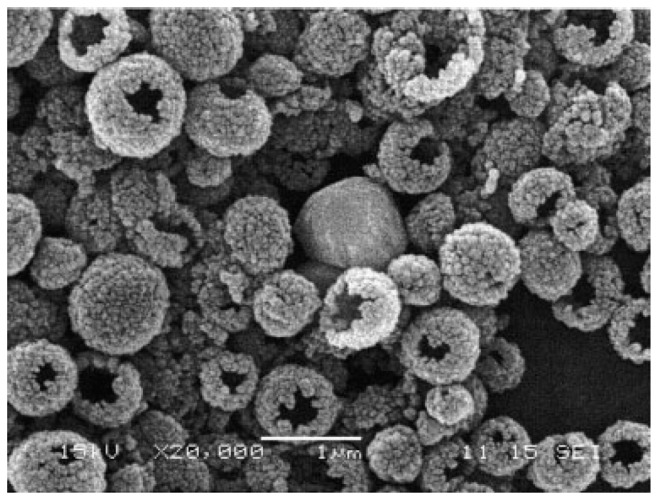
SEM micrographs of CaCO_3_ ‘hollow closed’ microspherical particles obtained in the presence of a triblock copolymer PEG750-*b*-[(P(O)(H)O(CH_2_)_6_]_17_-*b*-PEG750 for concentration of modifier 3 g·L^−1^. Reprinted with permission from [53]. Copyright (2005) Wiley-VCH Verlag GmbH & Co.

The effect of mPEG750-*b*-[(P(O)(H)O(CH_2_)_6_]_17_-*b*-mPEG750 and mPEG2000-*b*-[(P(O)(H)O(CH_2_)_6_]_17_-*b*-mPEG2000 triblock copolymers on crystallization of CaCO_3_ was studied by Penczek and coll. in 2005, the formation of microsherical and ‘hollow closed’ microspherical (Figure 7) crystallites was detected [53]. 

**Figure 8:**
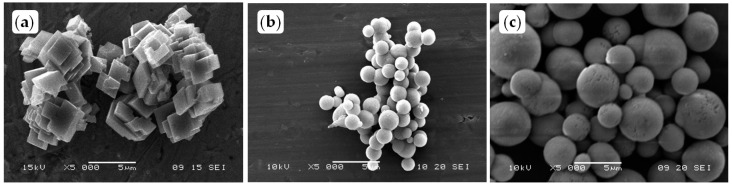
SEM images of CaCO_3_ particles obtained (**a**) in the absence of polymeric modifier; (**b**) in the presence of Na-PEP; (**c**) in the presence of phosphorylated glycerol. Magnification 5000×. Reprinted with permission from [40]. Copyright (2009) Taylor & Francis Group, LLC.

As shown by Jerome and coll. [85], the presence of block copolymer mPEG_5000_-*b*-(PEPA)_16_ has led to the formation of porous CaCO_3_ microspheres with uniform size distribution, the best results were achieved when using supercritical carbon dioxide (scCO_2_) technology. The marked difference between PEPA and the products of H_3_PO_4_/glycerol polycondensation is clearly seen in Figure 8 that demonstrates the morphology of the CaCO_3_ particles obtained in the absence and in the presence of PCPAs, in particular, sodium poly(ethylene phosphate) (Na-PEP) [40].

#### 3.2.3. Hybrid Nanoparticle Formation by Polyphosphodiesters

Magnetite nanoparticles, functionalized by PCPA (Scheme 20a) containing P–OH and P–OEt substituents in 1:9 ratio, exhibited a bimodal distribution with 18 and 113 nm particle diameters, in contrast with NOs stabilized by oleic acid and catechol-functionalized PPEs [33]. 

**Figure 9:**
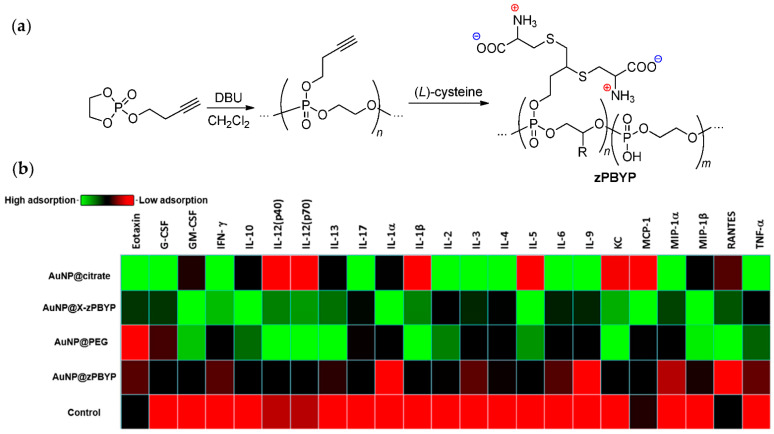
(**a**) Synthesis of zPBYP copolymer; (**b**) Cytokine adsorption on the coated AuNP formulations, calculated on the basis of apparent concentrations of cytokines measured after incubation with NPs and subsequent rinsing to remove unadsorbed material, as compared to the concentrations in a solution containing no NPs. The figure is the heatmap showing the concentrations of the various cytokines. Reprinted with permission from [102]. Copyright (2019) American Chemical Society.

As was mentioned in Section 2.3.4, in 2019 Wurm and coll. described polymerization of but-3-yn-1-yl ethylene phosphate, followed by thiol-ene click reaction with (*L*)-cysteine [102]. The resulting copolymer zPBYP (Figure 9a) was used for coating of Au nanoparticles (AuNPs, citrate-coated AuNP@citrate were used as a starting material) with a formation of AuNP@zPBYP, followed by cross-linking of cysteine fragments (AuNP@X-zPBYP). When compared with each other and with PEGylated AuNP@PEG, AuNP@X-zPBYP showed the highest cytokine adsorption (Figure 9b). 

### 3.3. Biomedical Applications of Polyphosphodiesters

Due to the presence of –P(O)(OH)– fragments, polyphosphodiesters exhibit such properties as acidity (and therefore ability to form salts with metal cations and organobases) and bone affinity (as a natural consequence of the similarity of the phosphodiester fragment in PCPA and PO_4_^3−^ anion in bone mineral). Polyphosphodiesters are also subjected to hydrolysis, which makes them potentially valuable biodegradable polymer materials for various clinical applications. However, the studies of polyphosphodiesters have not yet made it beyond the laboratory despite many promising results obtained.

#### 3.3.1. Polyphosphodiesters and Cell Viability/Metabolism

It is clear that biomedical application of PCPAs requires the absence of adverse impact of PCPAs on a living organism. Given the relatively high hydrolytic lability of P–OR bond, main-chain and side-chain PCPAs should be considered separately. For polyphosphodiesters, the possible impact may be caused by the formation of alkyl phosphates (RO)P(O)(OH)_2_ that represent both relatively strong acids and organic compounds with acutely under-researched properties. A wide range of polyphosphodiesters have been studied to date by different cytotoxicity tests. Differences in the used cell cultures, experimental methods and conditions do not allow us to summarize the data in the table, and just a brief summary of the facts will be given here. 

**Figure 10:**
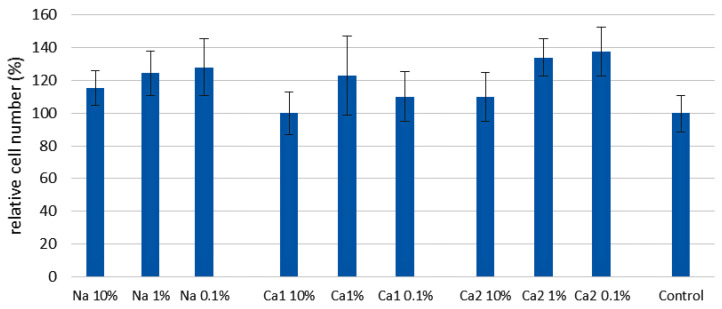
The results of 7-day cell adhesion and proliferation experiments for the solutions of PEPA metal salts diluted by the factors of 1000, 100 and 10. The starting solutions of the salts were prepared in phosphate/metal molar ratios of 1:1 (Na-PEP, Ca1-PEP) and 2:1 (Ca2-PEP). The initial concentration of phosphate groups was 0.443 mmol/g. The percentage of the cell viability relative to control (additive-free plates) is presented. Reprinted with permission from [125]. Copyright (2019) MDPI.

In both HeLa cells and RAW 264.7 mouse macrophage cells, no cytotoxicity was detected for Na-PEP over the range of concentrations from 5 to 1250 μg/mL [90]. The effect of Na-PEP (in comparison with inorganic polyphosphate) on viability of the osteoblastic MC3T3-E1 cells was studied by Iwasaki et al. [83], the cell compatibility of Na-PEP was better than that of inorganic polyphosphate, visible decrease in cell viability was observed only from the 10 mg∙mL^−1^ concentration of Na-PEP. Cholesterol-initiated PEPA/poly(EtOEP) copolymer demonstrated no hemolysis activity or cytotoxicity against MC3T3-E1 cells [80,89]. No cytotoxicity was detected during experiments on adipose-tissue-derived multipotent mesenchymal stem cells (ADSCs) adhesion and proliferation in the presence of Na and Ca salts of PEPA [125] (Figure 10).

Cytotoxicity test of the random PEPA/EtOEP (H/E) copolymers (see Scheme 18) with mouse osteoblastic cells (MC3T3-E1) showed that the adverse effect of polyphosphoester ionomers on cell viability was significantly lower than was that of pamidronate (H_2_NCH_2_CH_2_C(OH)(PO_3_HNa)_2_), e.g., the IC_50_ of copolymer with H_21_E_79_ composition was approximately 200 times greater in mass concentration than that of pamidronate. Note the IC_50_ value of H_21_E_79_ was tripled by sodium salt formation [88].

The 1,2-PPPA with a pK_a_ 2.3 showed no toxicity to COS-7 and MRC-5 cells up to a concentration of 5.4 mg/mL [95].

Hyperbranched 3,6-dioxaoctan-1,8-diyl bis(ethylene phosphate)-based polymers (see Scheme 27) did not significantly affect the cell viability of the MDA-MB-231 cancer cells [108], thus providing the purity of the further experiment on loading and release of the anticancer drug (see Section 3.3.5).

**Figure 11:**
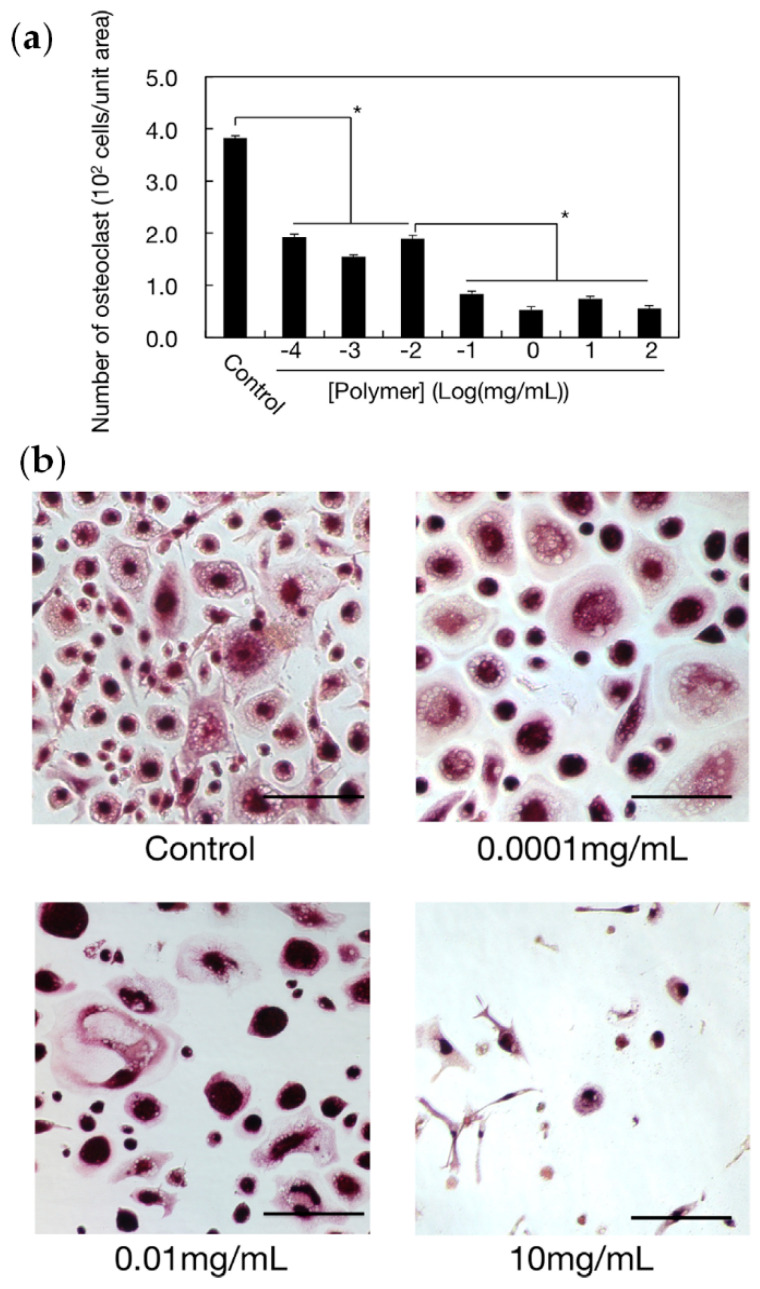
(**a**) Densities of adherent osteoclasts on bovine bone slices after incubation with Na-PEP for 24 h (* *p* < 0.005); (**b**) Optical micrographs of adherent osteoclasts on a bovine bone slice after cultivation with Na-PEP for 24 h. Scale bars represent 100 μm. Reprinted with permission from [99]. Copyright (2015) Wiley-VCH Verlag GmbH & Co.

A very important finding of the Iwasaki’ group was that Na-PEP did not trigger any change in osteoblast cell viability; however, the polymer diminished human osteoclasts and their ability to resorb bone at concentrations as low as 10^−4^ mg∙mL^−1^ [99] (Figure 11). This was the first report on using PPEs for selective inhibition of human osteoclast functions, indicating high potential of polyphosphodiesters as an effective polymer prodrug for osteoporosis treatment.

Since some polyphosphodiesters have a similar backbone structure to TAs, which makes up the cell walls of Gram-positive bacteria, Iwasaki and coll. synthesized a copolymer of Na-PEP and 2-(but-3-yn-1-yloxy)-1,3,2-dioxaphospholane 2-oxide p(EP/BYP), which mimics TA (terminal C≡C fragment was then used for insertion of fluorescent fragments via azide-click reactions) [82]. Copolymers showed no cytotoxicity with RAW 264.7 mammalian macrophages up to 10 mg∙mL^−1^ concentrations. It was found that RAW 264.7 exhibited higher uptake of copolymers than L929 mammalian fibroblasts. It was shown that high-MW copolymer (*DP*_n_ = 127) led to the highest intracellular transportation and the least gene expressions of IL-6 and TNF-α. 

#### 3.3.2. Polyphosphodiesters and Cell Differentiation

**Figure 12:**
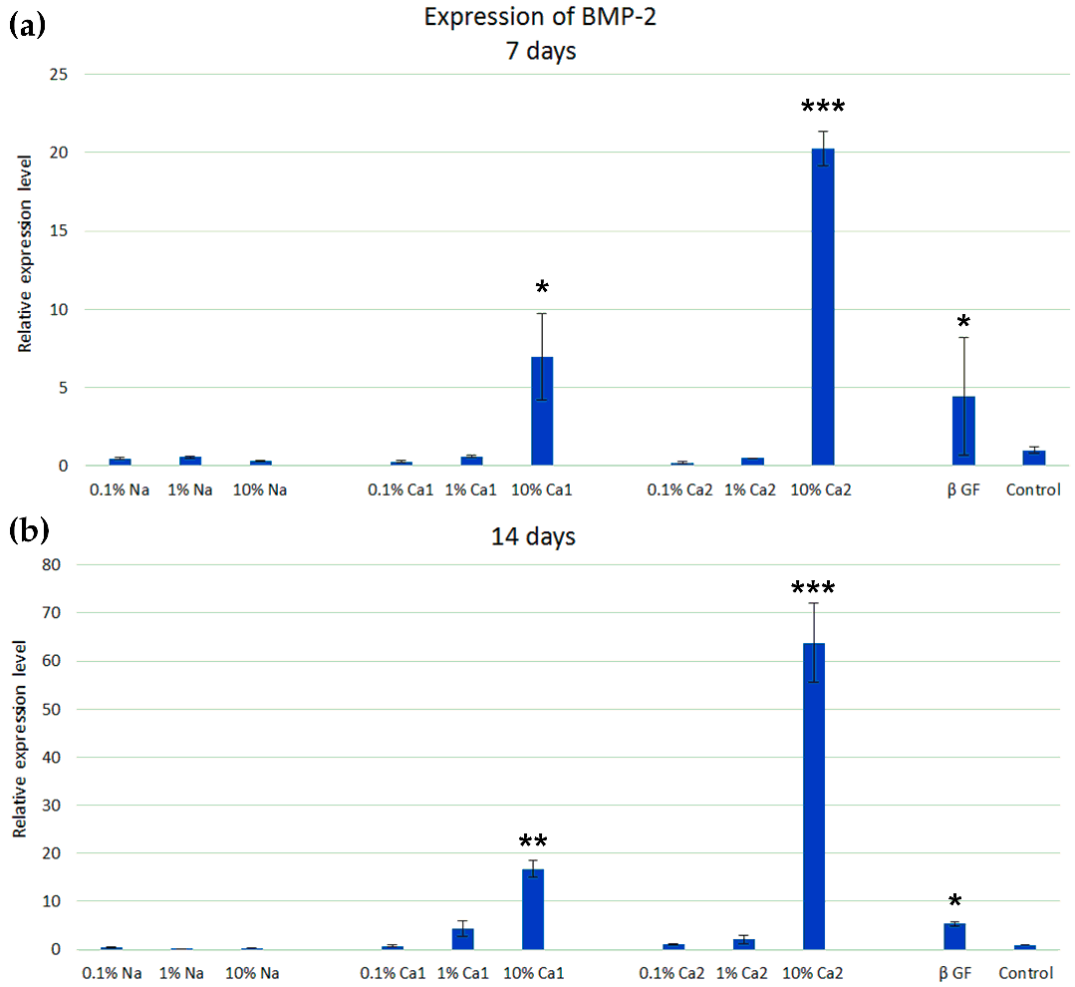
Differentiation of adipose-tissue-derived multipotent mesenchymal stem cells (ADSCs) with the solutions of PEPA salts Na-PEP, Ca1-PEP and Ca2-PEP of different concentrations, in the presence of β-glycerophosphate and in the control experiment in the absence of additives. The expression of the *BMP-2* gene in ADSCs on days 7 (**a**) and 14 (**b**). RT-PCR analysis. Statistical difference between a test group and control (* *p* < 0.05, ** *p* < 0.01, *** *p* < 0.001). Reprinted with permission from [125]. Copyright (2019) MDPI.

Nifant’ev et al. [125] reported that the calcium PEPA salts clearly induced osteogenic differentiation of the ADSCs, whereas the sodium salts were inactive within the margin of experimental error (Figure 12). Significant mineralization of the extracellular matrix during the cultivation of ADSCs with Ca-PEP was also detected.

**Figure 13:**
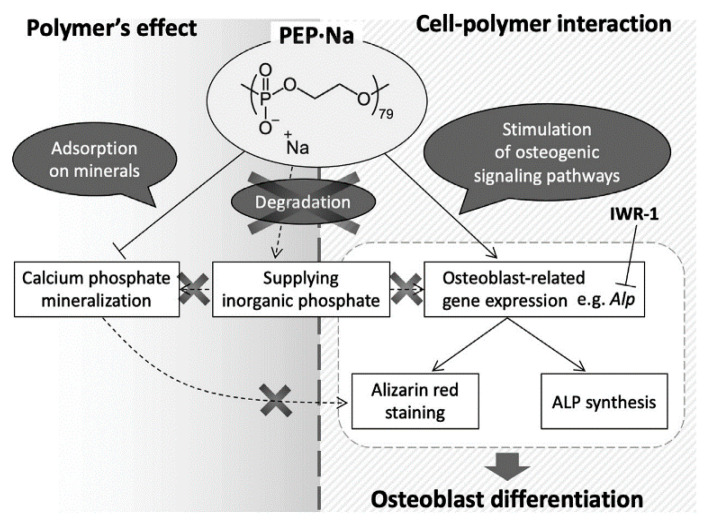
Schematic illustration of effects of Na-PEP on osteoblast differentiation of MC3T3-E1 cells. Reprinted with permission from [126]. Copyright (2021) Elsevier B. V.

More recently, Iwasaki and coll. performed a comprehensive study of the osteoblast differentiation with the use of Na-PEP and mouse osteoblastic cells MC3T3-E1 in a differentiation medium containing Na-PEP and poly(MeOEP) for comparison [126]. Substantial differentiation was detected for Na-PEP and described in Figure 13. 

#### 3.3.3. Polyphosphodiesters and Nucleic Acids, Proteins and Other Substances in the Body 

Polymers for biomedical applications should possess long-term stability in the bloodstream and should effectively minimize the interaction of the nanocarrier and blood components, e.g., poly(ethylene glycol) functionalization (PEGylation), so-called ‘stealth coating’, can prevent recognition by the reticuloendothelial system, thus preventing preliminary elimination of nanoparticles from the bloodstream and providing prolonged periods of circulation. It is thought that PEG can reduce non-specific protein adsorption and thereby confer a ‘stealth effect’. However, as demonstrated by Wurm and coll. [127], both PEG and poly(EtOEP), pre-exposed to plasma proteins, exhibit a low cellular uptake, whereas those not pre-exposed showed high non-specific uptake. In this way, the stealth effect still requires a *specific* adsorption of clustering proteins (apolipoprotein J). However, whether polyphosphodiesters have shown a stealth effect, is still an open question. 

For the development of bone-targeting polymeric prodrugs and other materials for bone surgery and tissue engineering it is essential that Na-PEP is inert toward thrombin, as shown by Iwasaki’ group, the adsorption of Na-PEP on thrombin-immobilized sensor was not observed [83].

In model experiments, both hyperbranched polymer’s (see Scheme 27) nanogel and PEG-6000 showed very limited bovine serum albumin (BSA) adsorption [108]. When studying bis(methacrylate)-based hydrogel (see Scheme 22), full hemocompatibility and the absence of the protein absorption from the coagulation cascade were demonstrated [104].

DNA complexation of Na-PEP was studied by Iwasaki and coll. [128] who studied the effects of molecular crowding with Na-PEP on the thermodynamics of DNA duplexes, triplexes and G-quadruplexes. Thermodynamic analysis demonstrated that Na-PEP significantly stabilized the DNA structures. At lower polymer concentrations, the stabilization was attributed to a shielding of the electrostatic repulsion between DNA strands by the sodium ions of Na-PEP. At higher polymer concentrations, the DNA structures were entropically stabilized by volume exclusion, which could be enhanced by electrostatic repulsion between phosphate groups in DNA strands and in Na-PEP. Additionally, increasing Na-PEP concentration resulted in increasing enthalpy of the DNA duplex but decreasing enthalpy of DNA G-quadruplex, indicating that the polymers also promoted dehydration of the DNA strands [128]. These results allowed us to elucidate the mechanisms involved in stabilizing DNA structures.

#### 3.3.4. Biocompatibility and Inflammatory Effect of Polyphosphodiesters

Despite their importance for biomedical applications, tissue biocompatibility and ability to cause inflammation remains mostly unexplored for polyphosphodiesters. The tissue response of polymer (see Scheme 27) nanogel after intramuscular injection was studied in C57BL/6J mice [108]. Histological analysis revealed no visible inflammatory reaction at the injection site after 7 days, which was comparable to muscle samples receiving PBS injections.

#### 3.3.5. Bone Affinity of Polyphosphodiesters and Their Prospects for Bone Surgery

This aspect of the use of PCPAs had been recently reviewed by Iwasaki [5]. Here, we will only briefly list and discuss some interesting and new results. 

As shown by Iwasaki and coll., random PEPA/EtOEP (H/E) copolymers (see Scheme 18) were able to be absorbed on the hydroxyapatite (HAp) surface [88]. Increases in the acid (H) content in copolymer resulted in higher values of adsorption, and sodium salt of H_21_E_79_ absorbed by one and a half times more effective than corresponding polyacid. In addition, these copolymers have inhibited HAp formation and resorption. Cholesterol-containing random PEPA/poly(EtOEP) copolymers of the similar structure significantly improved the affinity of the DOPC vesicles to calcium deposits generated by MC3T3-E1 cells [89].

Iwasaki and coll. also proposed sodium salts of PEPA as a new polymeric candidate material with an affinity to HAp and bone slices [80]. The affinity of Na-PEP nanoparticles to HAp was not suppressed by the presence of Ca^2+^ or low-pH conditions, which promote bone resorption by activated osteoclasts [80]. The BSA/Ch-Na-PEP complexes are well adsorbed onto HAp even in the presence of BSA (55 g/L) [81]. A bright and illustrious experiment, demonstrating high bone affinity of Na-PEP, was conducted by Iwasaki’ group with the use of Na-PEP copolymer, containing minimal amount of Cyanine 5 Azide (Cy5Az)-containing side groups [83]. Seventy-five hours after the intravenous injection of Cy5Az and Na-PEP-Cy5Az, qualitatively different fluorescence distributions were detected: no/weak signals for Cy5Az, but in the latter case fluorescence signals from bones located near the surface were significant (see Figure 4 in [83]). These findings enable us to consider that various types of polymeric prodrugs for bone disease treatment can be designed based on Na-PEP.

Copolymer p(EP/BYP) (see Section 3.3.1) was modified by thiol-yne click reaction with HS(CH_2_)_6_SCH_2_CH(Me)C(O)O(CH_2_)_2_OP(O)(O^−^)O(CH_2_)_2_NMe_3_^+^ [129]. The bacterial anti-attachment effects of the polymer-immobilized HAp materials were investigated via the adhesion of *S. mutans*. Because of its strong attachment to the HAp surface as a result of the anionic content, Na-PEP copolymer exhibited high bacterial anti-attachment efficacy.

In 2015 [100], Iwasaki and coll. proposed the use of Na-PEP covered PLGA microspheres (prepared using the water-in-oil-in-water emulsion solvent evaporation method from PLGA with *M*_w_ of 7–17 kDa with 1:1 lactate/glycolate ratio), α-tricalcium phosphate (α-TCP), castor oil, and water for the preparation of particle-stabilized self-setting emulsions with different component ratios, CPC-P0 (0/30/35/35), CPC-10 (10/30/30/30) and CPC-20 (20/30/25/25). After cement setting (24 h) and thermolysis at 600 °C, mesoporous materials were obtained, while PLGA microparticles resulted in the formation of an interconnected macroporous structure in the set cements which promoted extensive invasion of MC3T3-E1 cells. 

We believe that, due to proven high affinity of the polyphosphodiesters to HAp and bone mineral, the development of composite materials based on biodegradable polymers and bioresorbable calcium phosphates is a very important and promising scientific direction. Similar composites are well studied and have already been implemented in dentistry for side-chain PCPAs; however, in the case of polyphosphodiesters the research has been fragmentary at best.

#### 3.3.6. Drug Delivery and Drug Release with the Use of Polyphosphodiesters

Wang et al. reported the results of the study of binding and release of lysozyme (5% initial loading) with the use of 1,2-PPPA/Ca^2+^ hydrogels [95]. The release of lysozyme followed zero-order kinetics after an onset of 1 h and completed in 22 h with no burst release. 

**Scheme 31:**
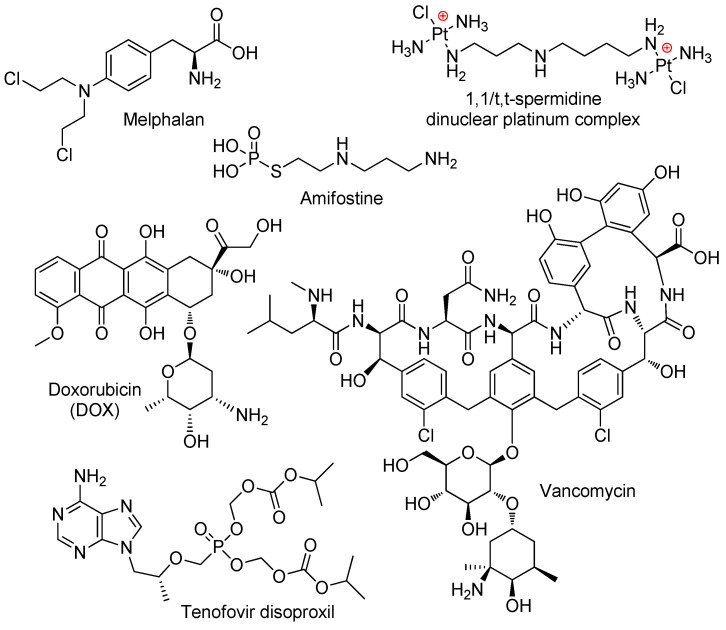
Structural formula of the medications studied in drug delivery and release with the use of polyphosphodiesters.

**Figure 14:**
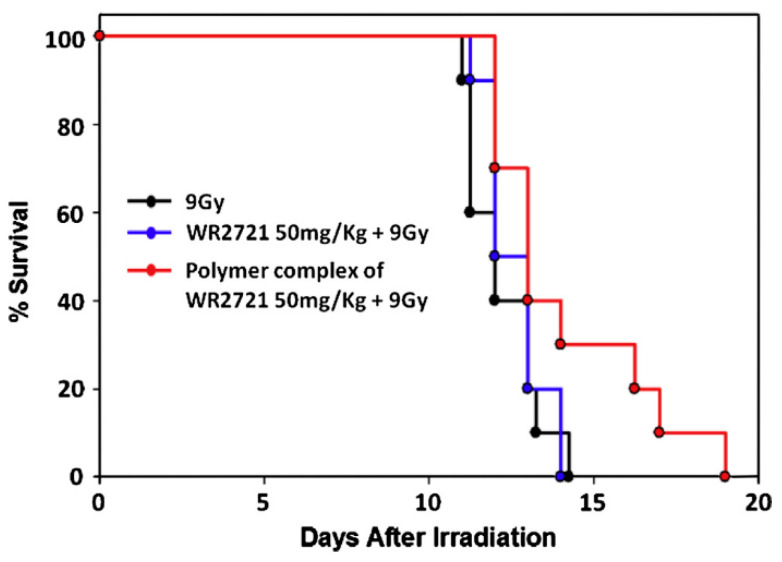
Overall survival of mice treated with 9 Gy whole body irradiation. The Kaplan–Meier plot shows percent survival plotted as a function of days after irradiation. Three groups of 10 mice each were exposed to 9 Gy WBI alone (black circles), WR2721 ip at a dose of 50 mg/kg 30 min prior to WBI (blue circles), or the polymer complex of WR2721 at an ip dose of 50 mg/kg 30 min prior to WBI (red circles). Reprinted with permission from [130]. Copyright (2014) Elsevier B. V.

PCPAs represent polyanions at physiological pH and are therefore capable of electrostatic binding with organic bases, including drugs. However, the ability of PEPA-containing polymers to act as a carrier of drugs with basic functional groups have not been studied in depth. In the early work of Troev et al. [27], similar interaction between –[O(CH_2_CH_2_O)_12_P(O)(OH)]*_n_*– and cytoprotective reagent amifostine (Scheme 31), applied in the radiation or cyclophosphamide cancer treatment. In this work, the formation of the adduct was confirmed by FT-IR spectroscopy. In further studies, the effect of the use of polymer complex was examined in comparison with amifostine alone [130], visible positive effects can be seen in Figure 14.

When studying formulations of the same polymer with cytostatic drug melphalan (Scheme 31) it was shown that covalent bonding of the drug is preferable in comparison with cation/anion complexation [131].

This research team also studied adduct of the copolymers with similar structure with dinuclear 1,1/t,t-spermidine platinum complex (Scheme 31), obtained via covalent bonding to poly(oxyethylene H-phosphonate)s applying the Athertone–Todd reaction [25]. The cytotoxic activities of the adducts were determined in a panel of five chemosensitive and one cisplatin-resistant tumor cell lines, but they were found to be less active than the prototype dinuclear complex.

Adducts of tenofovir disoproxil (TFD) with block copolymers mPEG-*b*-PEPA were recently studied by Nifant’ev et al. as candidates for developing a long-acting and controlled-release formulations for anti-HIV therapy using the model of experimental HIV infection in vitro (MT-4/HIV-1_IIIB_). Judging by the values of the selectivity index, TFD exhibited an up to 14-fold higher anti-HIV activity in the form of mPEG-*b*-PEPA adducts [87].

Hyperbranched 3,6-dioxaoctan-1,8-diyl bis(ethylene phosphate)-based polymers (see Scheme 27) contains both –P(O)(OH)– and secondary/tertiary amine fragments and therefore have a relatively low capacity for complexation with organobases. The studies of the loading of doxorubicin (DOX, Scheme 31) at 10:1 nanogel/DOX weight ratio have showed only 4% value of the drug loading [108], and doxorubicin release in the absence of phosphodiesterase I was found to be unbalanced (32% after 24 h and 52% after 11 days). Anticancer efficiency of the nanogel/DOX formulation against MDA-MB-231 cells was lower in comparison with DOX. Experiments with fluorescein isothiocyanate-modified nanogels (average diameter 171 of nm) showed significant nanogel internalization in MDA-MB-231 cells.

In one of their recent publications [92], Nifant’ev and coll have studied and discussed fundamental questions dealing with biomedical prospects of PCPAs:

- compatibilization effect of copolymers, containing polyester and PEPA block, on formation and properties of polyester/HAp composites.
- influence of PEPA on drug absorption and release by polymer/HAp composite.

**Figure 15:**
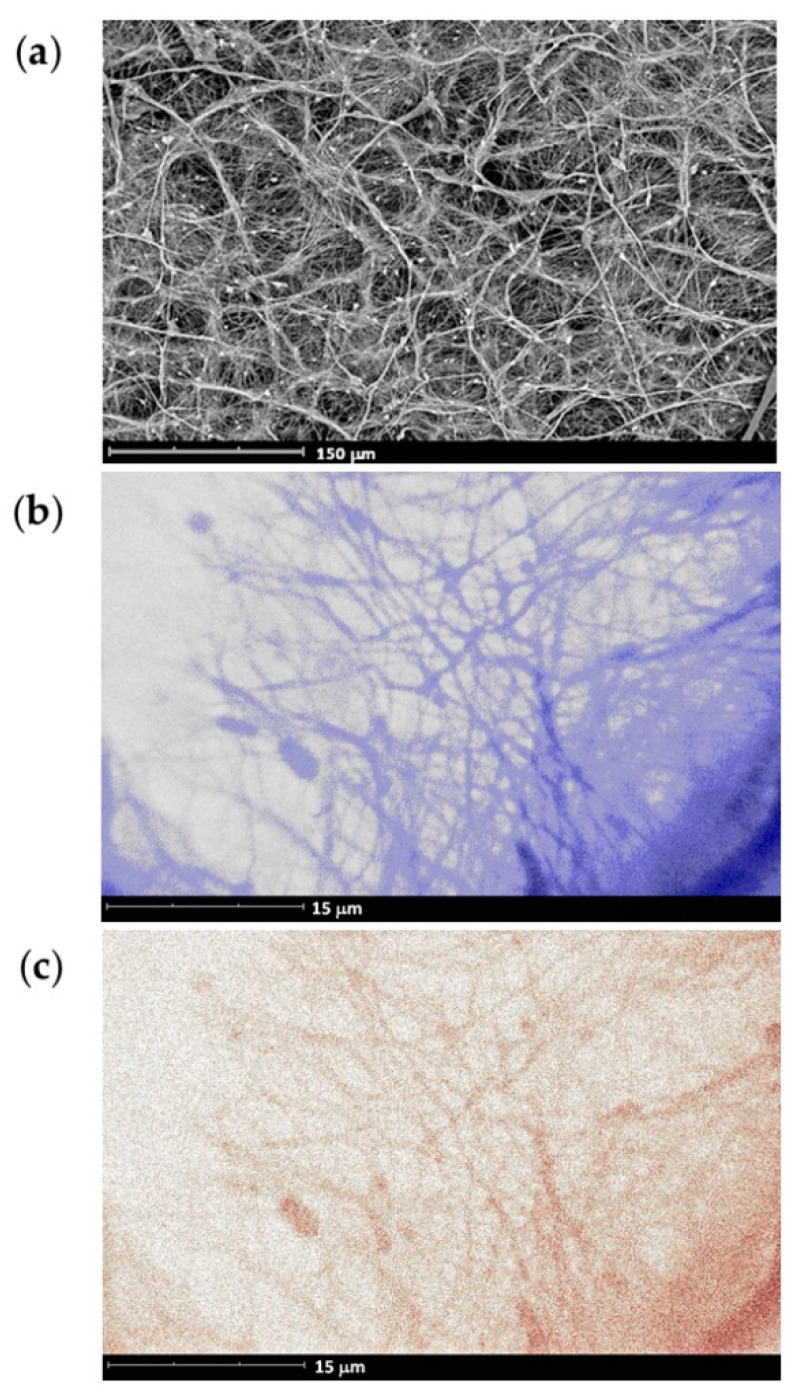
(**a**) SEM image of PCL/HAp composite ES fibers, prepared in the presence of 5% wt PCL-*b*-poly(*^t^*BuOEP); The distribution of (**b**) Ca and (**c**) N in ES fibrous film (SEM EDX mapping data). Reprinted with permission from [92]. Copyright (2021) MDPI.

It was demonstrated that BnO-(εCL)_118_-*b*-(tBuOEP)_6_, after deprotection with a formation of PEPA block, stabilize colloidal dispersion of nano-sized HAp (50–100 nm long and 20–50 nm wide) in solution of poly(εCL) (*M*_n_ = 87.5 kDa, *Ð*_M_ = 1.46) in hehafluoroisopropanol. This stabilization allowed to use electrospinning (ES) for the formation of fibrous composite material without critical HAp aggregation (Figure 15a). Different methods of the addition of vancomycin (Scheme 31) were studied, and the best results were achieved when vancomycin was added into spinneret solution before ES molding (Figure 15b,c). The samples of the fibrous mats have demonstrated high activity against *St. aureus*. In this way, high efficiency of the main-chain PCPA-containing compatibilizers was demonstrated, which opens up prospects for their use in further development of polyester/Ca phosphate composites for different biomedical applications. 

### 3.4. Other Applications of Polyphosphodiesters

#### Polyphosphodiesters as Flame Retardants

**Figure 16:**
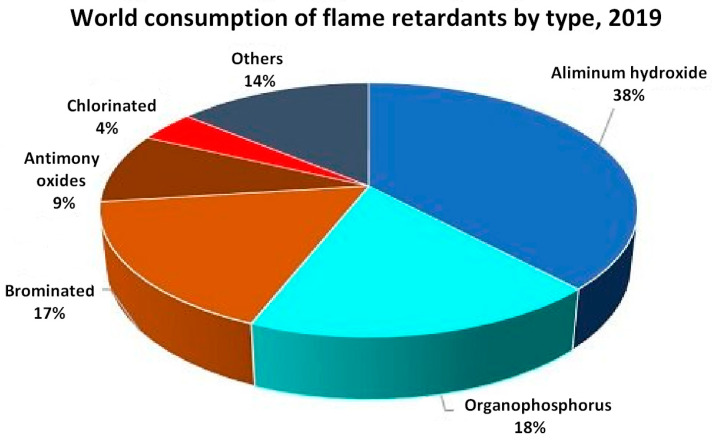
Global flame retardants (FRs) market categorized based on the chemistry [134].

Flame retardancy is one of the critical performance parameters to be considered in the design of polymers [132]. In industrial and academic applications, phosphorus-containing compounds play a crucial role in polymer flame retardants (FRs), as they are less harmful to the environment compared to the persistent and possibly bioaccumulating halogen-based flame retardants [133]. According to a 2019 market study [134], phosphorus-based compounds represent the third most used family of the FRs (Figure 16).

However, most of the organophosphate flame retardants represent triesters, not PCPAs. Wooley and coll. [90] proposed the use of the linear PEPA sodium salts as an alternative fire-retardant material due to high thermal stability and ultrahigh phosphorus and oxygen content (70 wt%), but it was a single work on this prospective theme. This was surprising, given the higher synthetic availability of PEPA and PEPA analogs, obtained by polycondensation method. It is very possible that results of Penczek’ group will find in this field a favorable ground for their application.

## 4. Conclusions

In our review, we tried to show all the diversity of the synthetic approaches to polyphosphodiesters and great potential of their applications. In our humble opinion, the consideration of polyphosphodiesters as a particular case of biodegradable polyesters [3,4,12,15] or tailor-made functional polyolefins [18,135] artificially and unjustifiably limit the assessment of these type of materials. The fundamental difference of the polyphosphodiesters from biodegradable polyesters are lower hydrolytic stability, higher biocompatibility, ability to deliver drugs with basic fragments, and bone mineral affinity. The capability of the polyphosphodiesters to demonstrate osteoinductive effect, as well as to form complexes with bases, provide obvious prospects for the further fruitful development of composite materials for bone surgery and dentistry, as well as drug delivery vehicles for different therapeutic purposes. The very idea of the synthesis of amphiphilic block copolymers, bringing together lipophilic block (providing micelle formation, or compatibilization with polyester in polymer/inorganic composite) and hydrophilic polyphosphodiester block (osteoinductive, osteoconductive, able to drug delivery) began to be realized only in the recent years. 

## Figures and Tables

**Table 1:** Synthesis of polyphosphonates and polyphosphates suitable for post-modification with a formation of PCPAs. The structures of the catalysts are presented in Scheme 13b.

Entry	Monomer	Catalyst	Reaction Conditions/Conversion, %	*M*_n_, kDa	*DP*_n_ ^a^	*Ð*_M_	Refs.
1	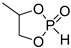	^i^Bu_3_Al	CH_2_Cl_2_, 20 °C	-	-	-	[65]
2	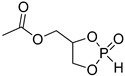	^i^Bu_3_Al	CH_2_Cl_2_, –20 °C, 6 h/80				[50]
3	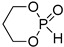	^i^Bu_3_Al	CH_2_Cl_2_, from 0 to 20 °C	90	740	-	[22,76]
4	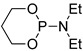	*^t^*BuOK	THF, 20 °C, several days/99	-	-	-	[77]
5	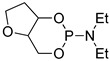	*^t^*BuOK	C_6_H_6_, 20 °C, several days/99	-	-	-	[72]
6	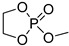	^i^Bu_3_Al	CH_2_Cl_2_, from –20 to 20 °C	30–100	-	-	[22]
		Et_2_Mg	CH_2_Cl_2_, from –20 to 20 °C	30–100	-	-	[22,67]
		DBU/TU	CH_2_Cl_2_, 20 °C, 15 min/83	9.2	68	1.17	[62]
		DBU/TU	CH_2_Cl_2_, 0 °C, 1.4 h/92	-	97	-	[78]
		Mg1	CH_2_Cl_2_, –20 °C, 5 min/99	9.5	70	1.35	[62]
		TBD/BnOH	CH_2_Cl_2_, –20 °C, 5 min/99	9.3	68	1.24	[62]
		TBD/BnOH	CH_2_Cl_2_, 1 eqiv. TMP, –20 °C, 5 min/99	6.4	47	1.13	[79]
		DBU/Cholesterol	CH_2_Cl_2_, 20 °C, 5 h/				[80,81]
7	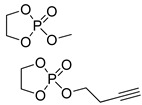	DBU/EtOH DBU/MeOH	9:1 comonomer ratio, CH_2_Cl_2_/– -	- -	38, 85, 127 73	- -	[82] [83]
8	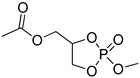	^i^Bu_3_Al	CH_2_Cl_2_, 0 °C	25	119	-	[50]
9	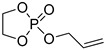	DBU/TU BnOH	toluene, 0 °C, 10 min/80	-	-	-	[84]
		DBU/TU mPEG_5000_	toluene, 0 °C, 10 min/80	7.5	16	<1.2	[85]
10	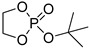	Et_2_Mg	C_6_H_6_, 40 °C, 10 h/80	25	139	-	[71]
		Mg1	CH_2_Cl_2_, 20 °C, 18 h	6.4	36	1.19	[62]
		Mg1	CH_2_Cl_2_, 20 °C, 18 h	–	63	–	[86]
		Mg2/mPEG_5000_	CH_2_Cl_2_, 20 °C, 30 h	3.6	13	1.45	[87]
			CH_2_Cl_2_, 20 °C, 30 h	8.1	49	1.48	[87]
11	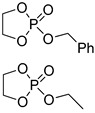	^i^Bu_3_Al	1:10 comonomer ratio, bulk/69.3	6.0–7.0	-	-	[70]
		TBD/BnOH TBD/ Cholesterol	5:95–20:80 comonomer ratio, toluene 4:96 and 17:83 comonomer ratios, CH_2_Cl_2_	9.5–11.9 4.6; 6.4	- -	1.45–1.62 1.3; 1.2	[88] [89]
12	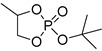	Et_2_Mg	C_6_H_6_, 40 °C, 10 h/90	25	139	n.d.	[71]
13	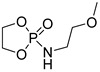	TBD/BnOH	CH_2_Cl_2_, 0 °C, 1 min/99	13	72	1.17	[90]
14	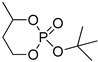	Et_3_Al/H_2_O	C_6_H_6_, 40 °C/50	-	-	-	[71]

^a^ Degree of polymerization.

## Data Availability

Not applicable.
